# TRAF3IP3 Induces ER Stress‐Mediated Apoptosis with Protective Autophagy to Inhibit Lung Adenocarcinoma Proliferation

**DOI:** 10.1002/advs.202411020

**Published:** 2025-03-11

**Authors:** Guang Zhao, Jun Qi, Fang Li, Haotian Ma, Rui Wang, Xiuyi Yu, Yufei Wang, Sida Qin, Jie Wu, Chen Huang, Hong Ren, Boxiang Zhang

**Affiliations:** ^1^ Department of Thoracic Surgery the First Affiliated Hospital of Xi'an Jiaotong University 277 West Yanta Road, Xi'an Xi'an Shaanxi 710061 China; ^2^ Department of Thoracic Surgery Sichuan Provincial People's Hospital: Sichuan Academy of Medical Sciences and Sichuan People's Hospital Chengdu Sichuan 610072 China; ^3^ Department of Dermatology Gansu Provincial Maternity and Child‐care Hospital (Gansu Provincial Central Hospital) Lan Zhou Gansu 730079 China; ^4^ Institute of Basic Medical Sciences Xi'an Medical University No.1 XinWang Road, Weiyang District Xi'an Shaanxi 710021 China; ^5^ Department of Cell Biology and Genetics School of Basic Medical Sciences Xi'an Jiaotong University Health Science Center Xi'an Shaanxi 710061 China; ^6^ Health Science Center Xi'an Jiaotong University Xi'an 710061 China; ^7^ Department of Thoracic Surgery the First Affiliated Hospital of Xiamen University Xiamen 361003 China; ^8^ Department of Radiation Oncology Shaanxi Provincial People's Hospital Xi'an Shaanxi 710061 China

**Keywords:** apoptosis, autophagy, ER stress, lung adenocarcinoma, STRN3, TRAF3IP3

## Abstract

TNF receptor‐associated factor 3 interacting protein 3 (TRAF3IP3/T3JAM) exhibits dual roles in cancer progression. While upregulated in most malignancies and critical for immune regulation. However, the specific effects and molecular mechanisms of TRAF3IP3 on the progression of lung adenocarcinoma (LUAD) remains poorly understood. This study reveals TRAF3IP3 is upregulated in several tumor tissues but exclusively decreased in LUAD and Lung squamous cell carcinoma (LUSC) tissues, consequential in a favorable overall survival (OS) in LUAD rather than LUSC. Herein, it is reported that TRAF3IP3 can suppress cell proliferation and promote the apoptosis rate of LUAD cells by inducing excessive ER stress‐related apoptosis. Importantly, TRAF3IP3 triggers ER stress via the PERK/ATF4/CHOP pathway, accompanied by stimulated ER stress‐induced cytoprotective autophagy in LUAD cells. Through IP‐MS analysis, STRN3 is identified as a direct downstream interactor with TRAF3IP3 and corroborated to regulate ER stress positively. Mechanistically, TRAF3IP3 facilitates the recruitment of STRN3 to the ER lumen through its transmembrane domain and fulfills its functional role in ER stress in an STRN3‐dependent manner in LUAD cells. Given its dual role in orchestrating ER stress‐associated apoptosis and autophagy in LUAD cell fate determination, the importance of TRAF3IP3 is highlighted as novel therapeutic target for LUAD treatment.

## Graphical abstract

Schematic diagram of the working model of TRAF3IP3 in the coordination of ER stress and autophagy related apoptosis in lung cancer cells.

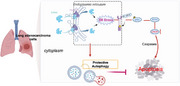

## 1 Introduction

Lung cancer remains the most commonly diagnosed and the leading cause of cancer death worldwide.^[^ ^1^ ^]^ Among all the pathological subtypes, lung adenocarcinoma (LUAD) accounted for the highest proportion, followed by lung squamous cell carcinoma (LUSC).^[^ ^2^ ^]^ As the most studied subtype, effective treatment such as targeted therapy or immunotherapy has dramatically improved survival in LUAD patients. Whereas drug resistance or lack of response may inevitably limit the effectiveness of such therapies.^[^ ^3^ ^]^ Thus, to solve the challenging issue and offer an innovative approach to the treatment of LUAD, it is imperative for researchers to investigate another powerful target.

TRAF3‐interacting protein 3 (TRAF3IP3), also known as tumor necrosis factor receptor‐associated factor (TRAF3)‐interacting JNK‐activating modulator (T3JAM), has dramatically intrigued our investigation. TRAF3IP3 was first identified in 2003 as an interactor to TFAF3 to activate the JNK pathway. It was found to be highly expressed in lymphatic tissue,^[^ ^4^ ^]^ and there is increasing research and exploration of TRAF3IP3 on the immune system. Previous studies have reported that TRAF3IP3 is required for B and T cell development and the maturation of Thymic natural killer T (NKT)2 cells, which could play a potential role in inflammatory and antitumor responses.^[^ ^5^, ^6^ ^]^ It was also reported to play a part in tumor progression.^[^ ^7^ ^]^ Patrick Nasarre et al. reported that TRAF3IP3 levels were substantially increased in the vasculature of breast cancer tissues and melanoma cells, suggesting its significant role in proangiogenesis and carcinogenesis.^[^ ^8^ ^]^ Moreover, TRAF3IP3 can promote glioma cell proliferation, migration, and invasion, and elevated TRAF3IP3 expression may serve as a potential biomarker for glioma prognosis.^[^ ^9^ ^]^ As to LUAD, the TRAF3IP3 expression profile or its role in LUAD proliferation and patient prognosis has not been investigated. Our current research demonstrated that TRAF3IP3 could inhibit the proliferation of LUAD cells both in vitro and in vivo while leaving the ability of migration and invasion unaffected, which differs from melanoma and glioma. Proteomic analysis was performed in LUAD cells, and the results revealed that the biological function of TRAF3IP3 may be involved in regulating endoplasmic reticulum stress‐associated apoptosis.

The endoplasmic reticulum (ER) is a vital organelle that exists in all eukaryotic cells where approximately more than a third of all cellular proteins are synthesized and folded.^[^ ^10^ ^]^ Given that high proliferative ability and requirements for protein synthesis are indispensable in cancer cells, various factors such as oxidative stress, genetic variation, nutrient deprivation, and metabolic abnormalities can disrupt the function of this organelle, which would cause endoplasmic reticulum stress (ER stress) that is featured by the accumulation of misfolded or unfolded proteins.^[^ ^11^ ^]^ Cells can rapidly respond to ER dysfunction through three adaptive ER stress pathways: Protein kinase R (PKR) ‐like ER kinase (PERK), inositol‐requiring enzyme 1α (IRE1α), and activated transcription factor 6 (ATF6), each of which directly or indirectly senses misfolded proteins through the molecular chaperone binding‐immunoglobulin protein (also known as GRP78),^[^ ^12^ ^]^ which could ultimately activate the UPR (unfolded protein response).^[^ ^13^ ^]^ When misfolded/unfolded proteins are overloaded in the ER, Grp78 separates from PERK, IRE1α, and ATF6, resulting in the activation of the three sensors. As the most well‐studied branch of the UPR, PERK can phosphorylate eukaryotic translation initiation factor 2α (eIF2α) to inhibit global protein translation, thus helping decrease the protein load in the ER, restoring the ER homeostasis, and simultaneously activating the downstream ATF4. However, PERK‐ATF4 signaling could contribute to apoptosis by upregulating the proapoptotic C/EPB homologous protein (CHOP) when unresolved or prolonged ER stress persists.^[^ ^12^, ^14^ ^]^ Once IRE1α is activated, IRE1α RNase splices a part of X‐box binding protein (XBP1) to generate XBP1s, which trans‐locates to the nucleus to activate the endoplasmic reticulum correlation (ERAD) and regulated IRE1‐dependent decay (RIDD), promotes protein folding and removes unfolded protein genes to maintain the homeostasis of the cell.^[^ ^15^ ^]^ Under ER stress, ATF6 activates and transfers to the Golgi apparatus to be cleaved by Site‐1 and Site‐2 proteases. The cleaved ATF6(N) transcription factor moves to the nucleus to increase the transcription of several UPR target genes, such as XBP1, GRP78, CRYAB, and VEGF, and enhances the ERAD pathway.^[^ ^16^ ^]^

Autophagy is a highly conserved intracellular degradation pathway in eukaryotes that can recycle superfluous protein and malfunctioning organelles.^[^ ^17^ ^]^ Autophagy's involvement in tumor progression is still controversial, as it can either promote or restrict tumor growth depending on the disease type and genetic environment. Furthermore, the final result of autophagy largely depends on the duration and intensity of the stimuli, and exceeding stimulation might lead to cytotoxic autophagy.^[^ ^18^ ^]^ A variety of signaling pathways, including class I PtdIns 3‐kinase (PI3K) AKT1 signaling, MTOR kinase, the response to endoplasmic reticulum (ER) stress, and the energy sensor AMP‐activated protein kinase (AMPK) pathway have been shown to be involved in the process of autophagy.^[^ ^19^ ^]^

Various studies have reported that prolonged or excessive ER stress is strongly correlated with metabolic disease,^[^ ^20^ ^]^ cancers,^[^ ^21^ ^]^ and inflammatory disease.^[^ ^22^ ^]^ Here, we show that TRAF3IP3 drives cell death of LUAD cells by causing excessive ER stress and promotes apoptosis through the PERK/ATF4/CHOP axis. The lethal impact of TRAF3IP3 is achieved through its interaction with STRN3 via the transmembrane domain, leading to the recruitment of STRN3 to the ER lumen. Furthermore, we demonstrated that ER stress induced by TRAF3IP3 results in cytoprotective autophagy, which could promote LUAD cell survival. Therefore, we postulate that combining TRAF3IP3 overexpression with pharmaceutical autophagy inhibitors will be an efficient treatment method for killing LUAD cells.

## 2 Results

### 2.1 Expression Profile of TRAF3IP3 and its Clinical Significance in LUAD Patients

**Figure 1:**
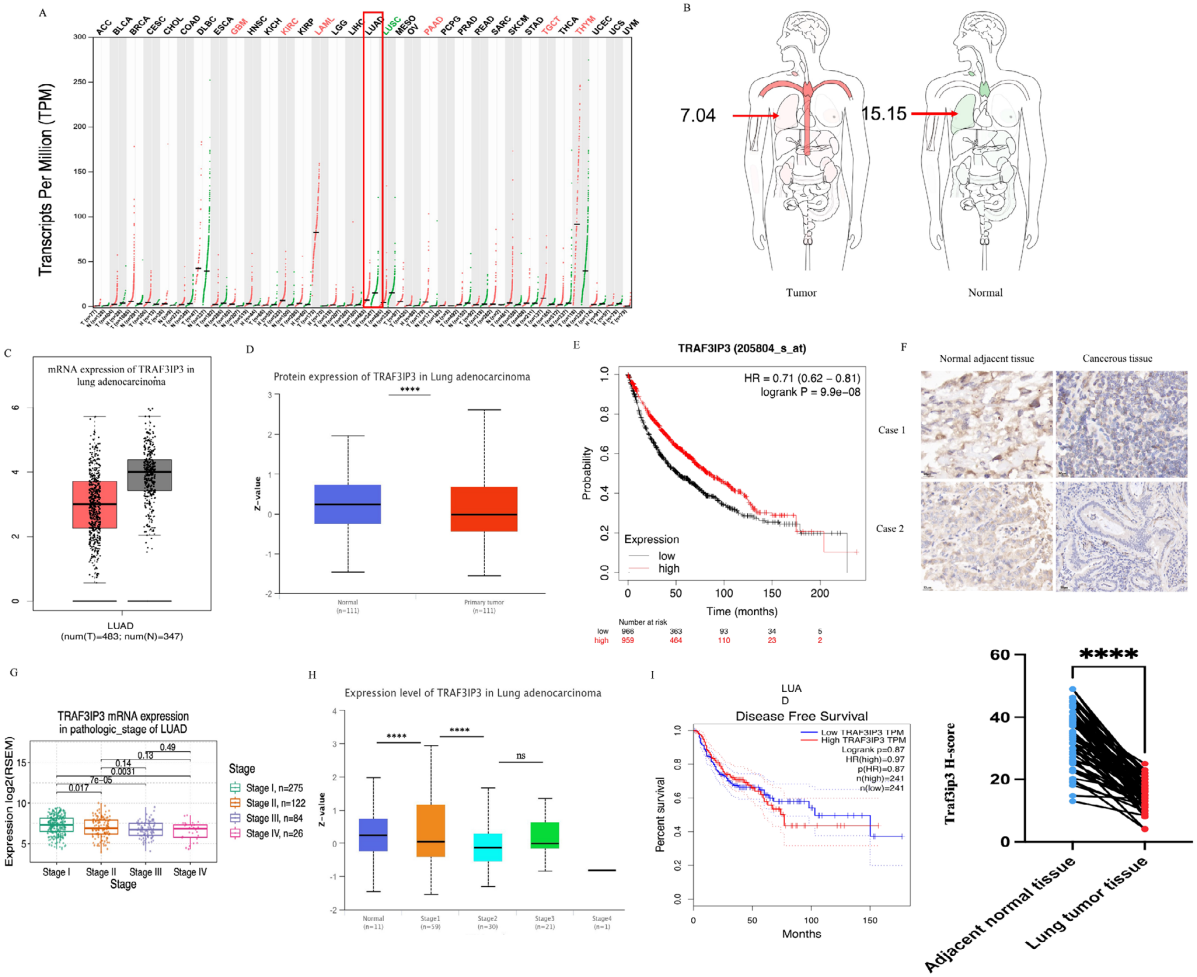
TRAF3IP3 is downregulated in lung cancer. A) The expression level of TRAF3IP3 was measured across 33 different tumors through the GEPIA online database. (http://gepia.cancer‐pku.cn/index.html). B) Bodymap obtained from GEPIA demonstrated that TRAF3IP3 has a lower expression in lung cancer than in normal tissues. C, D) Boxplots reveal that TRAF3IP3 mRNA (C) and protein (D) levels are downregulated in LUAD. E) Overall LUAD patient survival in patients with high and low expression of TRAF3IP3 was analyzed using the Kaplan‐Meier survival curve. The median OS of the two groups was compared by log‐rank test (*p* = 0.0042). F) Immunohistochemistry images demonstrating the levels of TRAF3IP3 in 63 pairs of normal adjacent lung tissues and LUAD tissues from patients diagnosed with LUAD. G, H) The mRNA level and protein level of TRAF3IP3 in LUAD patients with different tumor stages. I) Disease‐free survival of LUAD patients based on TRAF3IP3 expression level. ns: *p* ≥ 0.05, ***p < 0.001, ****p < 0.0001.

To investigate the functional importance of TRAF3IP3 in various human cancers, we initially examined the expression levels of TRAF3IP3 across different types of cancer in humans via the Cancer Genome Atlas (TCGA) RNA‐seq data accessed through GEPIA (Gene Expression Profiling Interactive Analysis). Among the 33 different types of human cancers, TRAF3IP3 mRNA expression was found to be down‐regulated only in lung adenocarcinoma (LUAD) and lung squamous carcinoma (LUSC). In contrast, Glioblastoma multiforme (GBM), Kidney renal clear cell carcinoma (KIRC), Acute Myeloid Leukemia (LAML), Pancreatic adenocarcinoma (PAAD), Testicular Germ Cell Tumors (TGCT), and Thymoma (THYM) showed significantly upregulated expression of TRAF3IP3, with no aberrant expression changes in the rest 25 types of cancer (**Figure** **1**A). An interesting result came to us that although the mRNA level of TRAF3IP3 is decreased in LUAD (LUAD: 7.04 versus normal: 15.15) (Figure 1B), it was not statistically significant (Figure 1C). However, Kaplan–Meier survival analysis revealed that a high level of TRAF3IP3 was significantly correlated with better overall survival (OS) in LUAD patients (Figure 1E). We then further analyzed the protein level of TRAF3IP3 in LUAD patients via the UALCAN database and discovered that TRAF3IP3 protein expression was markedly decreased in LUAD than in normal tissues, implying that TRAF3IP3 is likely to exert its biological function at the post‐translational level (Figure 1D). Patients with higher TNM stages (II‐III) showed lower TRAF3IP3 expression than patients with TNM stage I, as well as tumor grade, according to the TCGA dataset analysis. Hence, there was a negative association between TRAF3IP3 expression and TNM stage, tumor grade, and T stage in both mRNA and protein levels (Figure 1G,H; Figure S1A,B, Supporting Information). By analyzing the TCGA data on gender and ethnicity for overall survival, we found that male and African∖American ethnicity exhibiting high TRAF3IP3 expression levels, were associated with better survival (Figure S1C,D, Supporting Information). Subsequently, we detected the expression of TRAF3IP3 in tumor tissues versus matched normal tissues by conducting immunohistochemistry (IHC) analysis on 126 LUAD patients at our single center. Consistent with the TCGA dataset, TRAF3IP3 expression was significantly down‐regulated in human LUAD tissues, compared with those in adjacent noncancerous tissues (Figure 1F). On the other hand, 43 (62.3%) tumors and 15 (23.8%) paracancerous lung tissues showed low expression of TRAF3IP3 at the protein level. Collectively, TRAF3IP3 was significantly lower expressed in LUAD compared to adjacent lung tissues (Table S1, Supporting Information).

Furthermore, the relationship between TRAF3IP3 levels and the clinical pathological features in 126 LUAD and adjacent noncancerous tissues was assessed. Notably, the results demonstrated that TRAF3IP3 expression was negatively associated with tumor differentiation, lymph node metastasis, TNM stage and T stage (Table 1), consistent with above mentioned. Although TRAF3IP3 expression was shown to be lower in LUSC tissues, Kaplan‐Meier survival curves revealed no significant connection with OS and DFS in LUSC patients (Figure S1E–G, Supporting Information). These findings suggested that TRAF3IP3 might play a crucial role in LUAD.

Moreover, in six pairs of samples, TRAF3IP3 protein levels in tumor tissues were found to be lower than those in adjacent non‐cancerous tissues (Figure S1H, Supporting Information). TRAF3IP3 expression levels in normal lung epithelial cell lines (BEAS‐2B) and six NSCLC cell lines were also measured using RT‐qPCR and Western blotting. We discovered that TRAF3IP3 expression in LUAD cells was lower than in BEAS‐2B cells at both mRNA and protein levels, among which A549 and H1975 showed the lowest levels (Figure S1I, Supporting Information).

### 2.2 TRAF3IP3 Exhibits Tumor Suppressor Activities In Vitro and In Vivo

**Figure 2:**
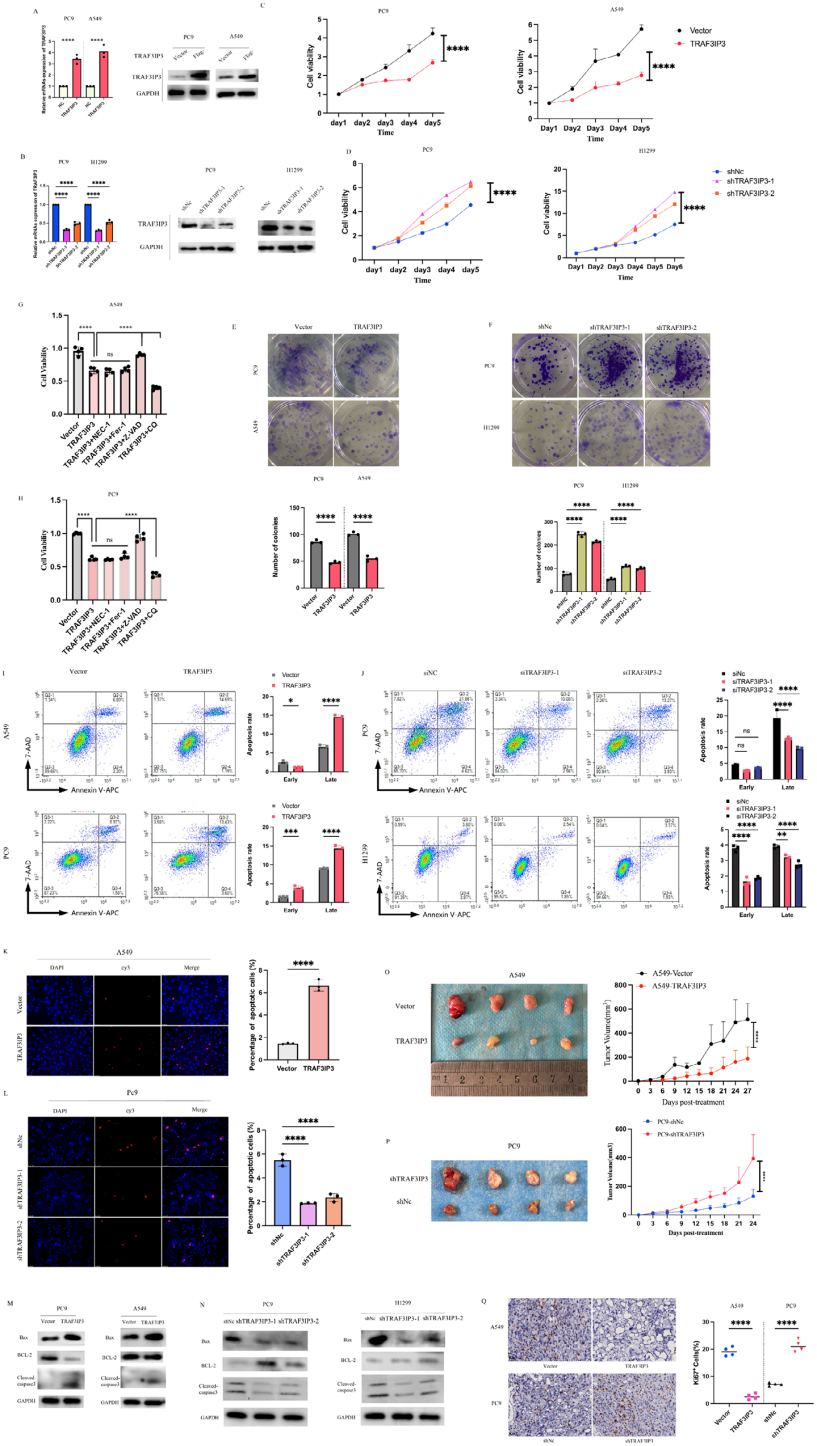
TRAF3IP3 regulates LUAD proliferation and apoptosis in vitro in vivo. A,B) TRAF3IP3‐overexpression cell lines (A549 and PC9) (A) and TRAF3IP3‐knockdown cell lines (PC9 and H1299) (B) were generated and validated using qRT‐PCR and western blotting analysis. n = 3. C,D) The proliferation rate of LUAD cells with overexpressing or knockdown TRAF3IP3 was determined by CCK8 assay. E, F) The effect of TRAF3IP3 overexpression and knockdown on the ability of LUAD cells to form colonies was determined using a colony formation assay. G,H) LUAD cell viability was detected by cck8 assay upon various cell death inhibitors. I, J) Flow cytometry analysis was used to determine the impact of overexpressing and knocking down TRAF3IP3 on apoptosis in LUAD cells. K,L) Cell proliferation detection of LUAD cells was measured by TUNEL assay. Scale bar, 50µm. M,N) The effect of overexpressing or knocking down TRAF3IP3 on the expression of apoptosis‐related molecules at the protein level in A549, PC9, and H1299 cells was determined using Western blot analysis, respectively. O,P) Images of the xenograft tumors from nude mice in each of the groups (n = 4); the tumor volumes of were measured every 3 days showed with tumor growth curves. (Student's t test). Q) IHC staining of ki‐67 was carried out in each group to measure cell proliferation capability with a scale bar set at 20 µm. A, B, E, F, I, J n = 3; C, D, G, H, O, P n = 4, Data were indicated as median ± SD, unpaired *t*‐test and one‐way ANOVA, ns: *p* ≥ 0.05, *p < 0.05, **p < 0.01, ***p < 0.001, ****p < 0.0001.

To investigate the functional significance of TRAF3IP3 in the progression of human lung adenocarcinoma, we conducted stable TRAF3IP3‐overexpression A549 and PC9 cell lines and TRAF3IP3‐knockdown PC9 and H1299 cell lines through lentiviral transfections, respectively. Empty vector and shNc were used as negative controls (NC), and the overexpression or knockdown efficiency was tested by qRT‐PCR and Western blotting (**Figure** **2**A,B, Figure S2A, Supporting Information). The CCK8 proliferation and colony formation assay revealed that TRAF3IP3 could markedly suppress tumor cell growth and colony frequency and size in A549 and PC9 cell lines, respectively (Figure 2C,E). Meanwhile, the cell growth and colony‐formation ability of PC9 and H1299 cells were dramatically increased in TRAF3IP3 knockdown cells (Figure 2D,F). To determine whether the anti‐tumor activity of TRAF3IP3 contributed to the induction of cell death, various cell death inhibitors were utilized. As shown in Figure 2G,H and Z‐VAD blocked TRAF3IP3‐driven cell death in A549 and PC9 cells. At the same time, Nec‐1 and Fer‐1 did not impact cell viability in such cells, indicating that the classical regulated cell death, apoptosis, was probably involved in TRAF3IP3‐driven cell death. Conversely, the autophagy inhibitor CQ was able to facilitate cell death induced by the overexpression of TRAF3IP3, prompting us to explore the role of autophagy in TRAF3IP3 function. As indicated by flow cytometric apoptosis assays, there was a marked elevation in the percentage of apoptotic cells in LUAD cells overexpressing TRAF3IP3 (Figure 2I). Conversely, the impact was reversed when TRAF3IP3 was suppressed (Figure 2J). Similar to the experiment above, ectopic expression or knockdown of TRAF3IP3 could markedly promote or reduce the apoptotic rate in LUAD cells than that of control cells (Figure 2K,L; Figure S2B, Supporting Information). In addition, the apoptosis‐related proteins were found to be evaluated. Consistently, TRAF3IP3 overexpression led to a reduction in the levels of the anti‐apoptotic protein BCL2 and an increase in the levels of apoptosis markers, cleaved‐caspase3 and Bax (Figure 2M,N).

To validate the function of TRAF3IP3 on LUAD tumor proliferation in vivo, subcutaneous xenograft tumor mouse models were established. A549 cells expressing vector or TRAF3IP3‐overexpression and PC9 cells expressing either a shRNA control (shNc) or shRNA against TRAF3IP3 (shTRAF3IP3) were injected subcutaneously into the axillary of nude mice, and the tumors were harvested after about 4 weeks. Figure 2O shows the xenograft tumor images and tumor volume plots against the tumor growth period. The comparison graph demonstrated that the tumor size of A549‐vector cells was significantly less than that of TRAF3IP3‐overexpression cells. In contrast, tumors generated from TRAF3IP3‐knockdown PC9 cells showed a greater size than those from control cells, as well as the tumor weight (Figure 2O; Figure S2C, Supporting Information). Additionally, IHC staining revealed that the number of Ki67^+^ cells was decreased in TRAF3IP3‐overexpression models and increased in TRAF3IP3‐knockdown models (Figure 2Q), which thoroughly validated the impact of TRAF3IP3 on LUAD growth. Nevertheless, according to wound healing and transwell assays, no significant change was observed in cell migration and invasion when TRAF3IP3 was either overexpressed or knocked down (Figure S2D,E, Supporting Information).

Briefly, these findings initially confirmed the anti‐cancer impact of TRAF3IP3 on the development and growth of LUAD cells, both in vivo and in vitro.

### 2.3 TRAF3IP3 Localizes in the Endoplasmic Reticulum and Promotes ER Stress

**Figure 3:**
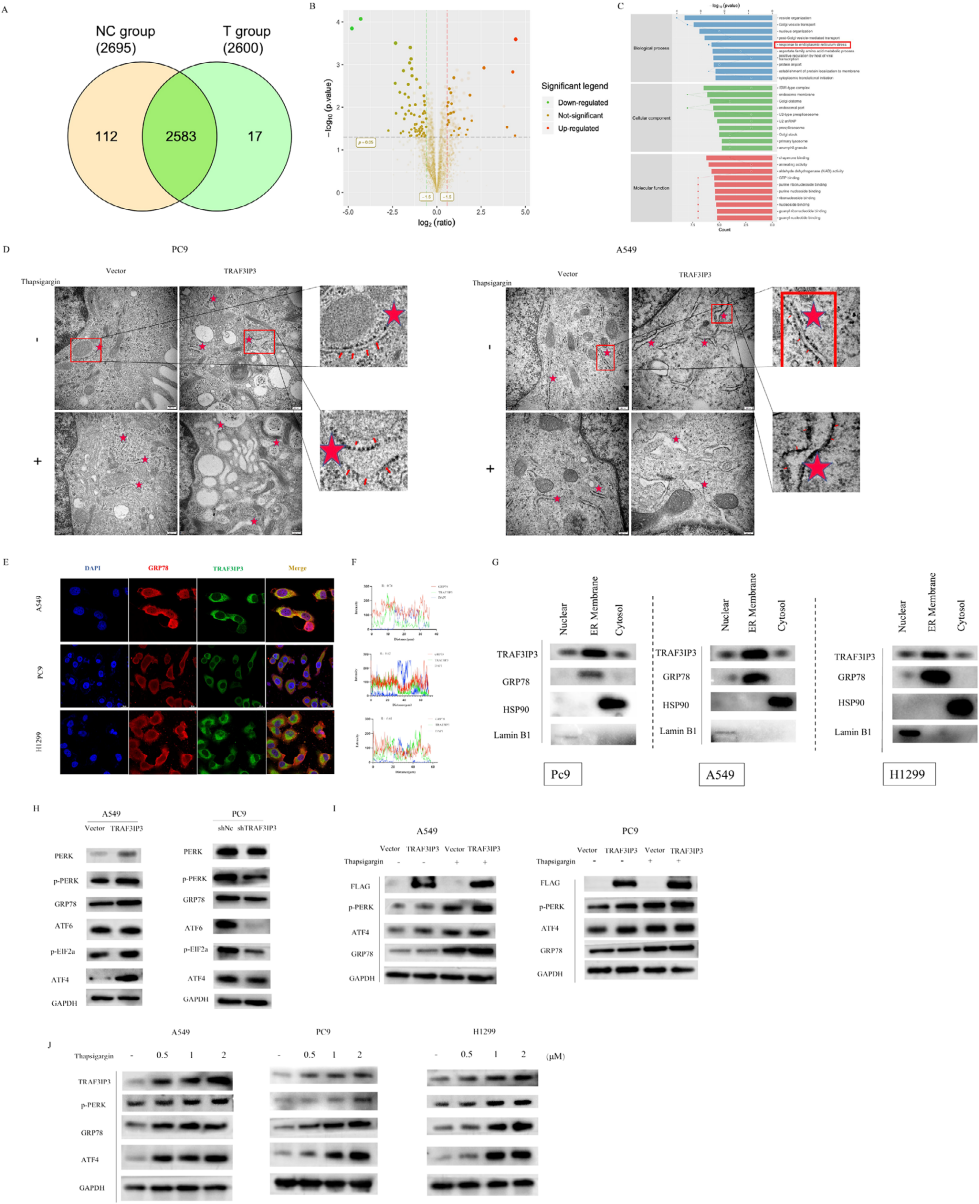
TRAF3IP3 is localized in the ER and involved in ER stress responses. A) Venn diagram showing differentially expressed proteins (DEPs) determined by LC‐MS/MS. A total of 2583 DEPs are shared between the two groups (2695 for the Flag‐vector and 2600 for the Flag‐TRAF3IP3 transfected cells). B) Volcano plot displaying DEPs between cells transfected with Flag‐vector and Flag‐TRAF3IP3, with red dots representing increased DEPs, green dots representing decreased DEPs, and brown dots indicating no significant difference (log2FC < −1). FDR < 0.05; *p* < 0.05. C) The gene ontology categories of DEPs in biological process, cellular component and molecular function. D) Endoplasmic reticulum of A549 and PC9 cells with stable overexpression of TRAF3IP3 with or without thapsigargin was examined by transmission electron microscopy (TEM); the ER was indicated by red pentagram. Scale bar, 200nm. E) Immunofluorescence co‐staining for TRAF3IP3 (green), ER stress marker GRP78 (red) and nuclear DNA (blue) in LUAD (A549, PC9, H1299) cells. F) The coincident curves showed the immunofluorescence colocalization level of GRP78 and TRAF3IP3 in the representative image. G) Immunoblot analysis was performed on proteins isolated from cytosolic, ER membrane, and nuclear fractions of A549, PC9, and H1299 cells using specific antibodies. H) Western blots showed ER stress‐related proteins, including p‐PERK, PERK, ATF 6, GRP78, p‐EIF2a, ATF4, and GAPDH protein levels. I) A549 and PC9 cells with TRAF3IP3 overexpression or with a control vector were treated with 1 uM thapsigargin for 18 h. Consequently, an immunoblot analysis was performed with the indicated antibodies. J) Western blots of TRAF3IP3, p‐PERK, GRP78, and ATF4 in thapsigargin‐treated A549, PC9, and H1299 cells.

To characterize the potential pathway and protein abundance changes of TRAF3IP3 that may be involved in LUAD progression, we performed proteomic analysis by Liquid chromatography‐mass spectrometry. The whole proteins of the corresponding samples were calculated according to the linear relationship between the standard curve and the absorbance OD562 nm, and a total of 2712 proteins were identified, with 2695 proteins in control vector‐expressing cells and 2600 proteins in TRAF3IP3‐overexpressing cells (**Figure** **3**A). The FDR of the peptide and protein levels were controlled at 0.01, and proteins with fold difference (ratio A/B >1.5, p‐value<0.05, unique peptide≥2) were defined as significant proteins. Finally, 97 proteins were identified as significantly differentially expressed in TRAF3IP3‐overexpressing cells, with 28 up‐regulated proteins and 69 down‐regulated proteins compared to negative control cells (Figure 3B; Figure S3A, Supporting Information). To seek insight into the functional relationships, Gene Ontology (GO) analysis was performed using the upregulated and downregulated DEPs to draw the functional annotation. Enrichment in “Golgi vesicle transport,” “response to endoplasmic reticulum stress,” and “Aspartate family amino acid metabolic process ” was found to be significant for the DEPs within GO biological process terms (Figure 3C). Since ER stress has been shown to be strongly associated with tumor cell proliferation and apoptosis by previous studies, we assume that TRAF3IP3 may play a role in regulating ER stress in LUAD cells.

To investigate the potential link between ER stress and TRAF3IP3, changes in the structure of subcellular organelles were first observed by transmission electron microscopy, followed by the verification of subcellular localization of TRAF3IP3 using immunofluorescence. As shown in Figure 3D, A549 and PC9 cells with TRAF3IP3‐overexpressing exhibit a marked dilatation of the endoplasmic reticulum lumen compared to vector‐expressing cells (marked with a red pentagram). Thapsigargin, a compound that can induce ER stress in various cells, was used to introduce ER stress in A549 and PC9 cells. The endoplasmic reticulum was found to be dilated following thapsigargin treatment in vector cells; in contrast, a more pronounced level of endoplasmic reticulum distension was observed in the TRAF3IP3‐overexpression cells (Figure 3D) (marked with a red pentagram). To our knowledge, GRP78 localizes in the ER, where it operates as a typical Heat shock protein 70 (HSP70) chaperone involved in the folding and assembly of proteins and acts as a master regulator of ER homeostasis. Immunofluorescence staining demonstrated co‐localization of TRAF3IP3 and GRP78 in A549, PC9, and H1299 cell lines (Figure 3E), implying a novel location of TRAF3IP3 in the ER, with a high coincidence curve exists between TRAF3IP3 (green) and GRP78 (red) (Figure 3F). In addition, subcellular organelle or membrane protein analyses of the three cell lines showed that TRAF3IP3 was expressed in endoplasmic reticulum membranes, nuclei, and cytoplasm, and was especially abundant in the endoplasmic reticulum (Figure 3G).

ER stress is a vital biological process that can respond to various situations, such as oxidative stress, genetic variation, or nutrient deprivation, to restore endoplasmic reticulum and intracellular homeostasis. Three ER sensors, IRE1, PERK, and ATF6, as well as GRP78 (ER chaperone protein) are involved in ER stress. To validate the effect of TRAF3IP3 on ER stress, we examined the ER‐related protein levels in A549 and PC9 TRAF3IP3‐overexpressed cell lines and PC9 and H1299 TRAF3IP3‐knockdown cell lines. The results revealed that overexpression of TRAF3IP3 increased the ER‐related proteins GRP78, PERK, phosphorylated PERK, and the PERK pathway downstream protein ATF4 and p‐eif2a. However, in contrast to shNc cell lines, ER protein levels were decreased in PC9 and H1299 TRAF3IP3‐knockdown cell lines (Figure 3H; Figure S3B, Supporting Information).

We then examined the changes in ER proteins during aggravated ER stress to validate the regulation of ER stress by TRAF3IP3 more reliably. The expression of ER‐associated proteins was found to increase in cells following treatment with thapsigargin compared with DMSO treatment. And there was a greater elevation in ER proteins following thapsigargin treatment in TRAF3IP3‐overexpressed lung cancer cells (Figure 3I). Along with the results of TRAF3IP3‐overexpressed cell lines, depletion of TRAF3IP3 caused decreased ER proteins compared to the shNc group, and the ER protein levels were comparable between shNc and shTRAF3IP3 cell lines following thapsigargin application (Figure S3D, Supporting Information). In addition, the promotional effects of TRAF3IP3 on ER stress were further confirmed by detecting the ER proteins under ER stress inhibitor, 4‐phenylbutyric (4‐PBA) treatment in A549 and PC9 TRAF3IP3‐overexpressed cell lines. The western blot analysis showed that the ER protein levels were diminished in the context of TRAF3IP3 overexpression when 4‐PBA was utilized, indicating a reduction of ER stress activation (Figure S3C, Supporting Information). Under conditions of ER stress, TRAF3IP3 overexpression was observed to result in increased ER protein levels in lung cancer cells, with opposite results in TRAF3IP3 knockdown lung cancer cells. Moreover, to examine the potential alterations in the expression of TRAF3IP3 in response to ER stress, we treated A549, PC9 and H1299 cells with varying concentrations of thapsigargin, as well as specific concentrations of thapsigargin for varying durations of exposure. We observed a significant elevation in the protein levels of TRAF3IP3, GRP78 and ATF4 (Figure 3J; Figure S3E, Supporting Information). The above results suggested that ER stress induces aberrantly increased TRAF3IP3 protein levels.

### 2.4 TRAF3IP3 Regulates ER Stress‐Related Apoptosis through PERK/ATF4/CHOP Axis

**Figure 4:**
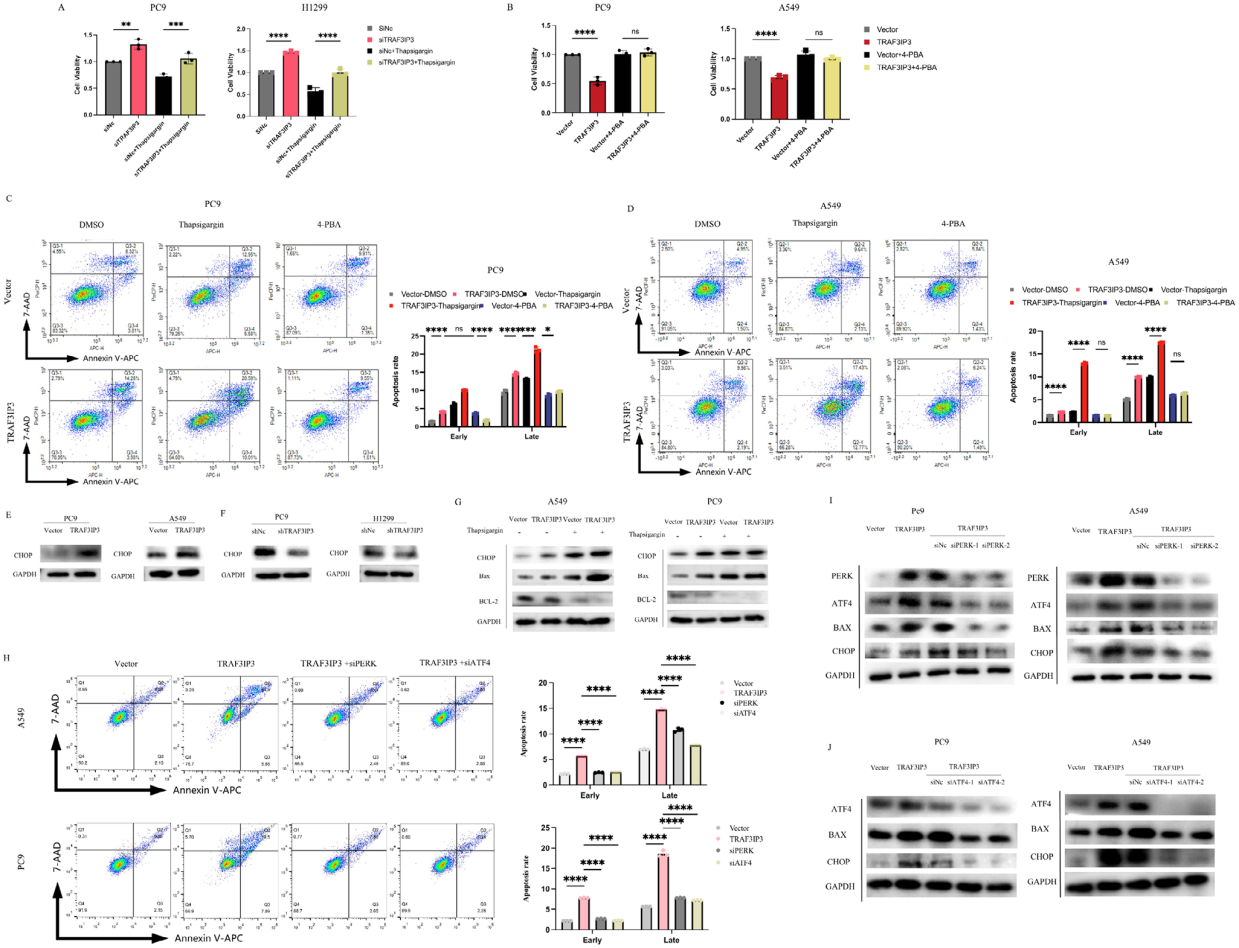
TRAF3IP3 stimulates ER stress‐related apoptosis through PERK/ATF4/CHOP axis. A) PC9 and H1299 cells were subjected to transfection with either Si‐control or Si‐TRAF3IP3, followed by exposure to 1 uM thapsigargin for 18 h, after which cell viability was assessed using the CCK8 assay. Data is shown as mean±SD of three independent experiments. **p < 0.01, ***p < 0.001, ****p < 0.0001. B) Flag‐Vector and Flag‐TRAF3IP3 A549 and PC9 cells were treated with 1 mM 4‐PBA for 18 h, followed by CCK8 assay to detect the cell viability. C, D) A549 and PC9 cells with vector or TRAF3IP3‐overexpression were treated with 4‐PBA (1 mM) or thapsigargin (1 uM) for 18 h, respectively, and then flow cytometry analysis was done to assess the apoptosis rate. Data is shown as mean±SD of three independent experiments. E, F) The impact of TRAF3IP3 on CHOP expression was evaluated by western blotting in A549, PC9 and H1299 cells. G) A549 and PC9 cells with TRAF3IP3 overexpression or with a control vector were treated with 1 uM thapsigargin for 18 h. Immunoblot analysis was performed to detect apoptosis‐related proteins including Bax, Bcl‐2 and CHOP levels. H) Depletion of PERK or ATF4 prevents TRAF3IP3‐induced apoptosis rate. Cells were transfected with Flag‐vector or Flag‐TRAF3IP3 plasmids for 24 h and then transfected with siRNA or PERK‐ and ATF4‐selective siRNAs and the apoptosis rate was determined by flow cytometry. I) Scramble siRNA or two different PERK‐selective siRNAs were transfected into A549 and PC9 cells overexpressing TRAF3IP3 for 48 h. The expression levels of ATF4, Bax and CHOP proteins were analyzed by immunoblotting. J) Scramble siRNA or two different ATF4‐selective siRNAs were transfected into A549 and PC9 cells overexpressing TRAF3IP3 for 48 h. The expression levels of Bax and CHOP proteins were analyzed by immunoblotting. A, B n = 4; C, D, H n = 3, unpaired *t*‐test and one‐way ANOVA, median ± SD, ns: *p* ≥ 0.05, *p < 0.05, ****p < 0.0001.

Various studies have verified the correlation between ER stress and apoptosis.^[^ ^23^ ^]^ The presence of unfolded proteins in the ER can be triggered by numerous factors, leading to the activation of the unfolded protein response (UPR) and, in cases of significant or prolonged stimulation, resulting in cell death.^[^ ^24^ ^]^ To explore whether TRAF3IP3 induces apoptosis via UPR processes, A549 and PC9 transfected with TRAF3IP3‐plasmid or vector‐plasmid and PC9 and H1299 transfected with siRNA targeting TRAF3IP3 or control siRNA were used. A CCK8 assay was applied to evaluate cell proliferation using the indicated treatments. The ablation of TRAF3IP3 was observed to promote the proliferation of PC9 and H1299 cells. Depletion of TRAF3IP3 led to an increased growth rate; in contrast, cell proliferation was inhibited following thapsigargin treatment on si‐TRAF3IP3 cells, resulting in a proliferation rate comparable to that of the siNc group (**Figure** **4**A). Correspondingly, the proliferation rate was suppressed in cells (PC9 and H1299) transfected with TRAF3IP3‐plasmids; under treatment with 4‐PBA, the TRAF3IP3‐overexpressing group showed comparable cell viability when compared to the vector group (Figure 4B). Furthermore, the cell viability of the cells transfected with TRAF3IP3 and treated with thapsigargin was significantly decreased (Figure S4A, Supporting Information). However, the thapsigargin‐treated group showed a modest increase in cell proliferation in shTRAF3IP3 cell lines in contrast to the DMSO‐treated group (Figure S4B, Supporting Information). Next, we attempted to check the apoptotic effect of TRAF3IP3 using the flow cytometry assay. The flow cytometry assay revealed that the overexpression of TRAF3IP3 increased the proportion of apoptotic cells in A549 and PC9 cell lines. Apoptosis was also triggered by thapsigargin treatment in control cells; nevertheless, thapsigargin treatment heightened the impact of TRAF3IP3 overexpression on cells undergoing apoptosis. Moreover, the process of apoptosis in the TRAF3IP3‐overexpression group was attenuated by 4‐PBA treatment, resulting in a comparable apoptosis rate to the vector group. At the same time, 4‐PBA treatment showed no impact on the apoptotic rate in the vector group (Figure 4C,D).

We conducted western blotting experiments to further corroborate the impact of TRAF3IP3 on apoptosis under ER stress. The activation of CHOP and cleaved‐caspase 3 are hallmarks of apoptosis in the presence of ER stress.^[^ ^25^ ^]^ The results showed that the CHOP level was stimulated when overexpressing TRAF3IP3, and it decreased obviously in TRAF3IP3‐knockdown cell lines (Figure 4E,F). TRAF3IP3 overexpression and thapsigargin treatment synergistically upregulated Bax and CHOP dramatically but downregulated bcl‐2 in A549 and PC9 cells (Figure 4G). The levels of Bax and CHOP were moderately reduced in the TRAF3IP3 overexpression group after treatment with 4‐PBA (Figure S4D, Supporting Information). However, treating the ShTRAF3IP3 group with thapsigargin resulted in a moderate increase in ER stress‐apoptotic protein levels compared to the shNc non‐treated group (Figure S4C, Supporting Information). In addition, after the addition of 4‐PBA, the expression of apoptotic proteins was comparable in the shNc and shTRAF3IP3 groups, both of which had reduced levels compared to those without the addition of 4‐PBA (Figure S4E, Supporting Information). These findings indicated that overexpressing TRAF3IP3 significantly increased ER stress‐mediated apoptosis in lung cancer cells, while depletion of TRAF3IP3 can reverse the ER stress‐mediated apoptotic rate.

Three transmembrane proteins are involved in response to ER stress: PERK, IRE1, and ATF6. Under ER stress, PERK could be phosphorylated and inhibit protein translation via phosphorylating eIF2α, followed by activating ATF4. Sustained PERK‐ATF4 signaling can lead to high levels of CHOP, inhibiting the anti‐apoptotic BCL‐2 and activating the pro‐apoptotic Bax, leading to mitochondrial apoptosis.^[^ ^26^ ^]^ The proteomic analysis results also suggested that TRAF3IP3 may function through the PERK pathway. To validate whether TRAF3IP3 induces ER stress‐linked apoptosis through the PERK pathway, siRNAs target PERK and the downstream proteins ATF4 were applied. We measured the frequency of apoptotic cells in A549 and PC9 TRAF3IP3‐overexpressing cells with simultaneous depletion of PERK or ATF4. Through flow cytometry analyses, either PERK or ATF4 knockdown could significantly rescue apoptosis triggered by TRAF3IP3 overexpression in lung cancer cells (Figure 4H). Consistently, silencing PERK with siRNAs led to a significant reduction in the levels of ATF4 and CHOP induced by TRAF3IP3 in A549 and PC9 cells, as well as to a down‐regulation of Bax. Likewise, a dramatic reduction in the expression of ER stress and apoptotic proteins induced by TRAF3IP3 was also observed upon silencing ATF4 with siRNAs (Figure 4I,J). Together, our data implies that the overexpression of TRAF3IP3 triggers ER stress and UPR activation in lung cancer cells. Under stress conditions, TRAF3IP3 could significantly enhance ER stress‐induced apoptosis through the PERK–ATF4–CHOP signaling pathway.

### 2.5 TRAF3IP3 Induces ER Stress‐Mediated Autophagy in LUAD Cells

**Figure 5:**
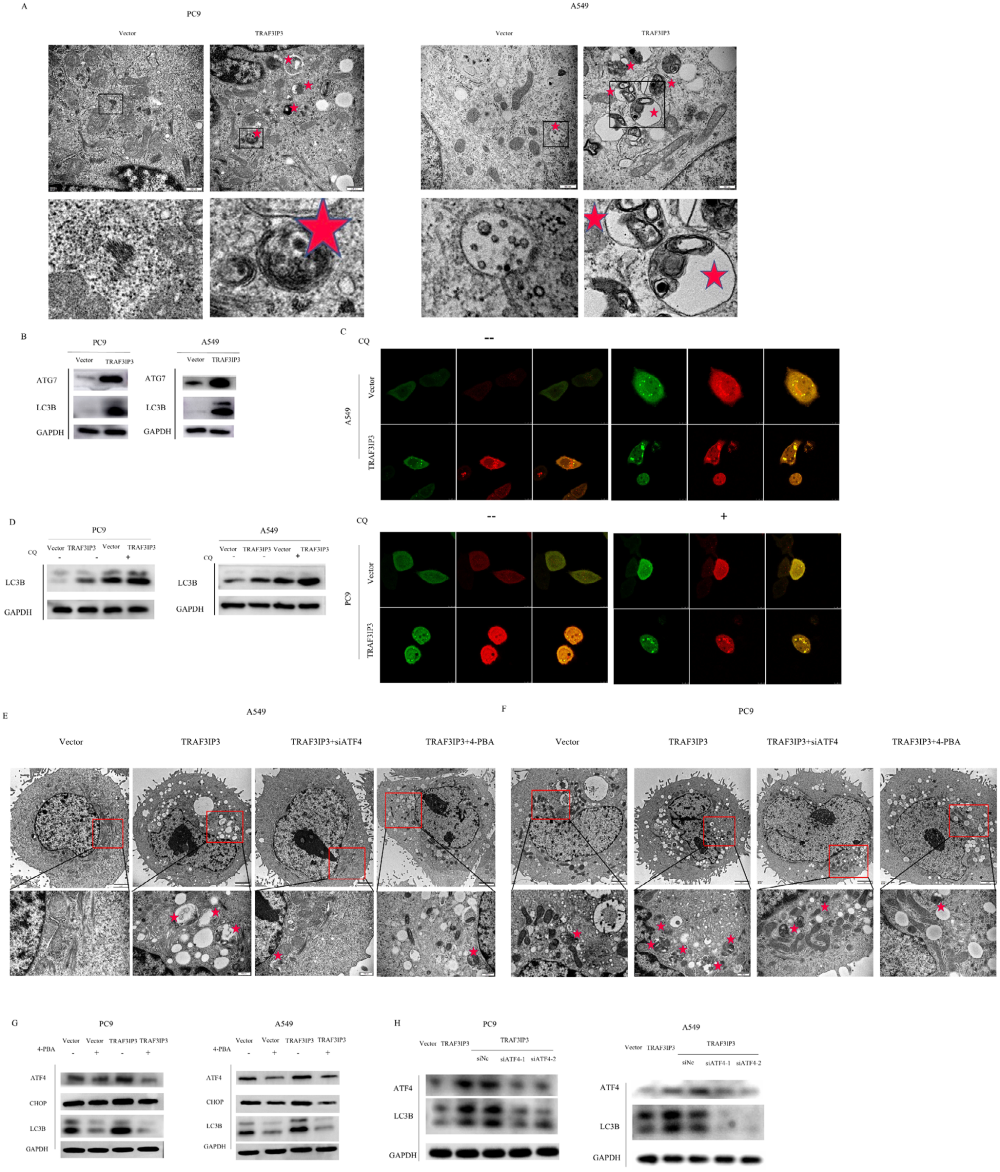
TRAF3IP3 induces ER stress‐mediated autophagy in LUAD cells. A) Autophagosomes of A549 and PC9 cells with stable overexpression of TRAF3IP3 were examined by transmission electron microscopy (TEM); the autophagosome was indicated by red pentagram. Scale bar, 500 nm. B) Western blotting was performed to detect autophagy‐related proteins (LC3B, ATG7) in TRAF3IP3‐overexpressing A549 and PC9. C) TRAF3IP3‐overexpressing A549 and PC9 with or without CQ treatment cells were transfected with GFP‐mRFP‐LC3 for confocal microscopic analysis to examine the expression of GFP and RFP. Three experiments were performed. Mean ± SD showed the number of autophagosomes (yellow dots) and autolysosomes (red dots) per cell. D) Western blotting analysis of the autophagic flux in A549 and PC9 cells overexpressing TRAF3IP3 and in their control cells. Rescue of LC3B degradation was achieved with the lysosomal inhibitor chloroquine (CQ). E,F) Autophagosomes of A549 or PC9 cells with stable overexpression of TRAF3IP3 were transfected with ATF4 SiRNA or treated with ER stress inhibitor 4‐PBA (1 mM for 24 h) and examined by TEM; the autophagosome was indicated by red pentagram. Scale bar, 500 nm. G) A549 and PC9 cells overexpressing TRAF3IP3 were treated with ER stress inhibitor 4‐PBA for 18 h, and then immunoblot analysis was done to measure the level of ATF4, CHOP, and LC3B expression. H) TRAF3IP3‐induced autophagy is prevented by silencing ATF4. Scramble siRNA or two different ATF4‐selective siRNAs were transfected into A549 and PC9 cells overexpressing TRAF3IP3 for 48 hours. The expression levels of LC3B proteins were analyzed by immunoblotting.

We have established the correlation between TRAF3IP3 and ER stress in the above experiments. Interestingly, we also stumbled across a lot of autophagosome formation under transmission electron microscopy when we examined the structure of the ER (**Figure** **5**A). A former study has also linked TRAF3IP3 to autophagy.^[^ ^6b^ ^]^ The conjugation of the soluble form of MAP1LC3B/LC3 to phosphatidylethanolamine and conversion to the autophagosome membrane‐associated form (MAP1LC3B‐II) is one of the hallmarks of autophagy. Overexpressing TRAF3IP3 significantly increased the ratio of LC3‐II to LC3‐I (Figure 5B). We also examined the expression of autophagy‐related 7 (ATG7), one of the autophagy‐related proteins, and found that the TRAF3IP3‐overexpression group exhibited increased ATG7 expression, while ATG7 and LC3 II/LC3‐I ratio decreased apparently upon TRAF3IP3 depletion (Figure 5B, Figure S5A, Supporting Information). To visualize autophagy flux, we transfected stable TRAF3IP3 overexpressed A549 and PC9 cell lines with LC3 lentivirus labeled by mCherry and GFP. Since GFP is sensitive to acidity, when autophagosomes fuse with lysosomes, GFP fluorescence is quenched, leaving only red fluorescence, and yellow puncta indicates the autophagosome or phagophore. LC3 dots and red puncta were increased obviously after TRAF3IP3 overexpression in LC3‐mCherry‐GFP‐labeled A549 and PC9 cells, implying that TRAF3IP3 can promote autophagy and increase autophagy flux (Figure 5C). As either an over‐induction of autophagy or reduced clearance of autophagic vesicles could contribute to the accumulation of lipidated LC3 and autophagosomes, we inhibited autophagy using chloroquine (CQ), which prevents the fusion of autophagosomes and lysosomes, to distinguish between these two possibilities and further elucidate the impact of TRAF3IP3 on the autophagic process. We found that autophagic vacuoles increased notably in TRAF3IP3‐overexpression cells after CQ treatment compared with the control cells (Figure 5A), and LC3‐II levels were further upregulated after CQ treatment (Figure 5D). Additionally, LC3‐II levels were decreased after treatment in TRAF3IP3‐knockdown cells with or without CQ treatment (Figure S5B, Supporting Information). It can be inferred from the results that the raised LC3 lipidation through TRAF3IP3 overexpression was at least partially due to increased autophagy induction. Consistent with what we've observed in A549 and PC9 cells, the number of autophagosomes increased after CQ treatment, which could be further increased by overexpressing TRAF3IP3, indicating that TRAF3IP3 might promote autophagic flux (Figure 5C). Autophagy can be activated upon ER stress to restore homeostasis in various cancer cells.^[^ ^13^, ^19^, ^27^ ^]^ To explore whether the autophagy induced by TRAF3IP3 was caused by the ER stress‐regulated PERK/ATF4/CHOP pathway, we used the TRAF3IP3‐overexpression A549 and PC9 cell lines for the experiments. Firstly, we asked whether TRAF3IP3‐induced autophagy was mediated by ER stress. TEM was applied to examine the autophagosomes in A549 cells with the indicated treatment. Results from TEM demonstrated that knocking down ATF4 or treating with 4‐PBA could majorly reverse the effect of TRAF3IP3 overexpression‐induced increased number of autophagosomes (Figure 5E,F). Overexpression of TRAF3IP3 led to a significant increase in ATF4, CHOP and LC3B levels; nevertheless, LC3B levels experienced a significant decrease following treatment with 4‐PBA (Figure 5G). Next, two different siRNAs targeting ATF4 were utilized to intercept the PERK/ATF4/CHOP axis, followed by LC3B expression measurement through a western blotting experiment. ATF4 silencing led to a marked decline in TRAF3IP3‐triggered upregulation of MAP1LC3B‐II (Figure 5H). There, these results potently suggested that activation of ER stress triggered by TRAF3IP3 mediates the induction of autophagy via the PERK/ATF4/CHOP axis.

### 2.6 Autophagy Plays a Protective Role in TRAF3IP3 Expression

**Figure 6:**
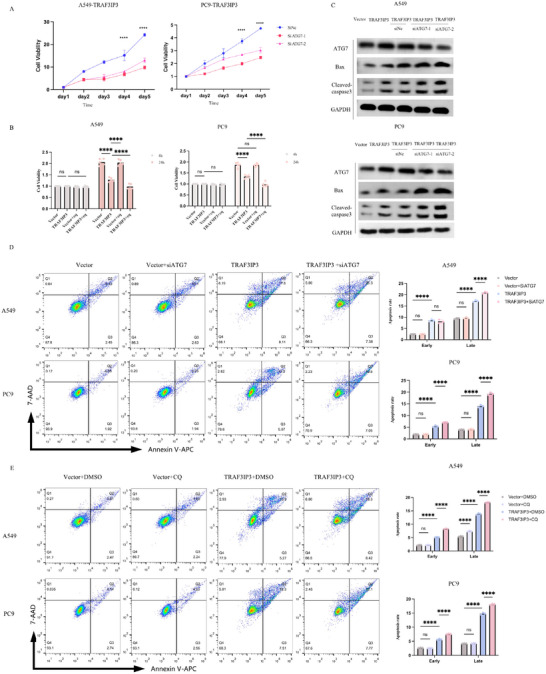
Autophagy plays a protective role upon TRAF3IP3 expression. TRAF3IP3‐overexpressing A549 and PC9 cells were transfected with anti‐ATG7 or control siRNA A) or treated with autolysosome inhibitor CQ and incubated for 4 and 24 h B), followed by CCK8 assays; C) the apoptosis‐related proteins expression level was analyzed by immunoblot. D) A549/PC9 cells were transfected with vector or TRAF3IP3 plasmid for 48h. The cells were then transfected with ATG7 siRNA for another 48 h, followed by flow cytometry analysis. E) A549/PC9 cells were transfected with vector or TRAF3IP3 plasmid for 48 h, then treated with CQ (20 µM) for 24 h followed by flow cytometry analysis. A, B n = 5; D, E n = 3, median ± SD, one‐way ANOVA, ns: *p* ≥ 0.05, ****p < 0.0001.

Autophagy is a highly conserved cellular process that acts as a scavenger to respond to diverse physical and pathological conditions. Notably, whether autophagy plays a protective or pro‐death role remains controversial. When we examined the endoplasmic reticulum by TEM, we also found a concomitant increase in the number of autophagosomes. Hence, we try to explore the role of autophagy in TRAF3IP3‐induced cell death in LUAD cells. A549 and PC9 stable cell lines overexpressing TRAF3IP3 were transfected with two ATG7 siRNAs or negative siControl to block the expression of ATG7. We then measured the proliferation rate of LUAD cells via CCK8 assay. The results showed that cell growth rates in the si‐ATG7 group were significantly lower than those in the siControl group (**Figure** **6**A). Subsequently, we investigated the impact of combination therapy with pharmacological blockade of autolysosome inhibitor CQ on cell proliferation in LUAD cells. Enhanced cell death was observed in TRAF3IP3‐overexpression cells following CQ treatment (Figure 6B), which validated the protective role of autophagy. In addition, the expression of Bax and cleaved‐caspase 3 was also analyzed by western blotting. The results demonstrated that Bax and cleaved‐caspase 3 levels exhibit a significant elevation after slicing ATF4 in TRAF3IP3‐overexpression cells compared with control cells (Figure 6C). Moreover, flow cytometry analysis showed a higher apoptosis rate of 27.88% or 26.85% in TRAF3IP3‐overexpression A549 and PC9 cells with ATG7 knockdown than that in TRAF3IP3‐overexpression cells (Figure 6D). We also evaluated the effect of CQ on apoptosis in A549 and PC9 TRAF3IP3‐overexpressing cells. Compared to DMSO, treatment with CQ further increased cell apoptosis in TRAF3IP3‐overexpressing cells. (Figure 6E). Collectively, the findings substantiate that autophagy exerts a protective influence on human NSCLC cells against the proliferative inhibitory effects mediated by TRIFAP3.

### 2.7 TRAF3IP3 Interacts with STRN3 and Promotes STRN3 Localization of Endoplasmic Reticulum

**Figure 7:**
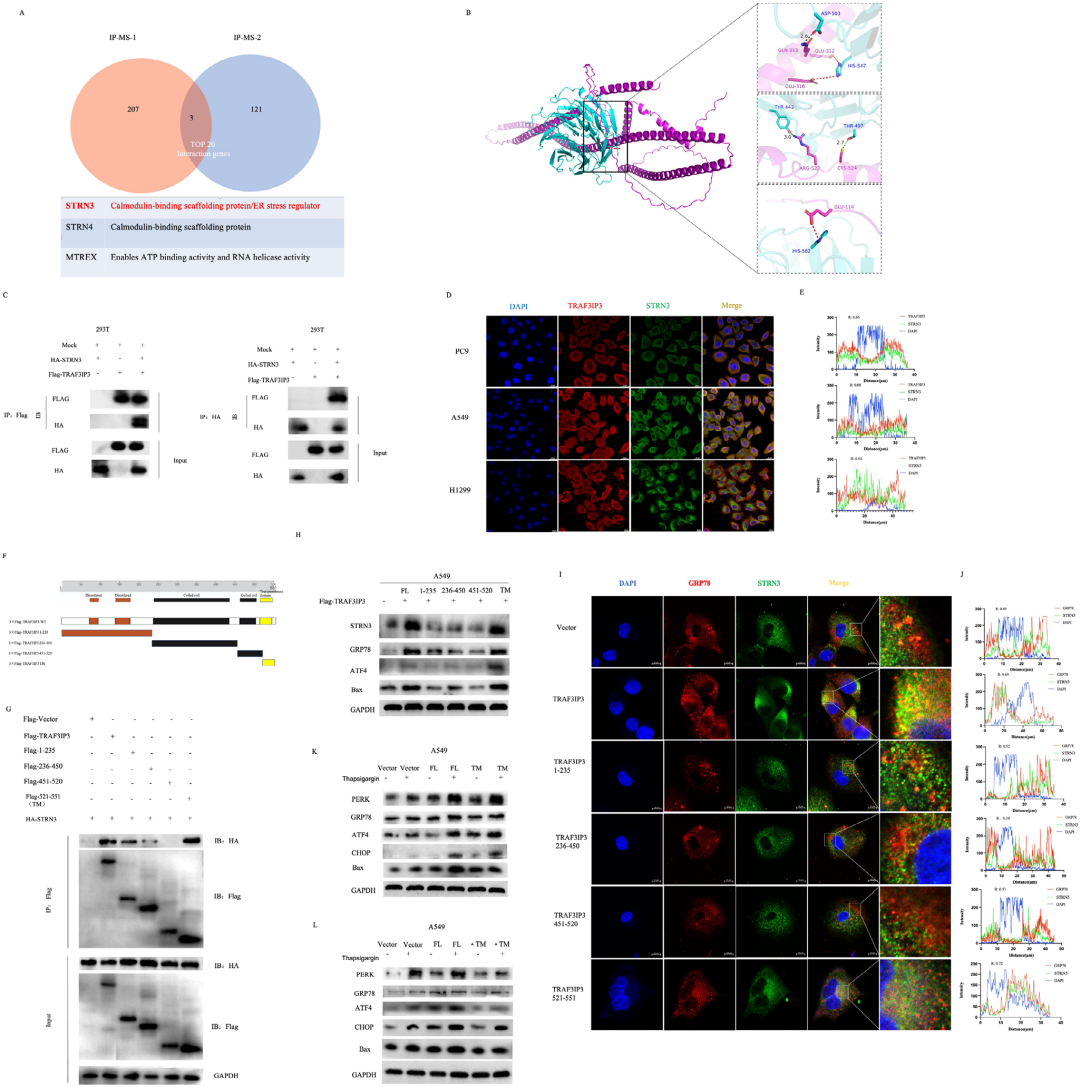
TRAF3IP3 interacts with STRN3 and promotes STRN3 localization of endoplasmic reticulum. A) STRN3 was identified as the binding partner of TRAF3IP3 by two replicate co‐IP mass spectrometry experiments. B) Molecular docking of TRAF3IP3 and STRN3. The purple molecule stands for TRAF3IP3, and the sky‐blue molecule stands for STRN3. C) Exogenous reciprocal coimmunoprecipitation (co‐IP) of TRAF3IP3 and STRN3 in 293T cells overexpressing Flag‐TRAF3IP3 and/or HA‐STRN3. D) Immunofluorescence staining of DAPI (blue), TRAF3IP3 (red) and STRN3 (green) in LUAD cell lines. E) The colocalization curves showed the colocalization level of STRN3 and TRAF3IP3 in the representative image. F) Schematic representation of wild‐type TRAF3IP3 and TRAF3IP3 deletion truncations. G Lysates from HEK293T cells transfected with HA‐tagged STRN3 and Flag‐tagged wild‐type TRAF3IP3 or TRAF3IP3 deletion truncations (1‐235, 236–450, 451–520, 521–551) were collected for coimmunoprecipitation and immunoblotting. H) A549 cell was transfected with indicated expression vectors, and STRN3, GRP78, ATF4, and Bax levels were detected by western blotting. I) Wild‐type TRAF3IP3 or deletion truncations were transfected into A549 cells, and confocal microscopy analysis of GRP78, STRN3 and DAPI was conducted. J) The colocalization curves showed the colocalization level of GRP78 and STRN3 in the representative image. K,L) A549 cell was transfected with control vector, wild‐type TRAF3IP3, specific containing transmembrane domain, and transmembrane domain‐deleted (rTM) plasmids; cell lysates were subjected to immunoblot with indicated antibodies.

To further explore the specific mechanisms by which TRAF3IP3 regulates ER stress‐apoptosis in LUAD cells, we performed liquid chromatography‐tandem mass spectrometry to investigate potential substrates that interact with TRAF3IP3. The protein of the A549 cell tagged with Flag‐TRAF3IP3 or Flag‐vector was harvested for IP collection with two repetitions. According to the IP‐MS analysis, the top ten molecules with the strongest binding affinity to TRAF3IP3 in the two experiments were selected and taken at the intersection to obtain the most likely target protein (**Figure** **7**A). Additionally, the protein interaction network of TRAF3IP3 was analyzed using STRING, which matched the results above (Figure S6A, Supporting Information). STRN3 stood out among the selected candidates because of its involvement in regulating ER stress, as shown in an earlier study.^[^ ^28^ ^]^ Through H‐docking, ten of 100 potential models were selected, among which model 1 showed the lowest binding energy scores (‐392.71) (Table S4, Supporting Information), suggesting that it is the most stable pose and the docking was successfully binding with significance. According to Figure 7B and Figure S6B (Supporting Information), the sky‐blue chain represents protein STRN3, and the purple chain represents TRAF3IP3. Our molecular docking results revealed that TRAF3IP3 interacts intensely with STRN3 via multiple domains. For example, GLU114, GLU316, and GLU312 of TRAF3IP3 form salt bridge with HIS562 and HIS547 of STRN3; CYS524, GLN313, and ARG522 of TRAF3IP3 could form hydrogen bonds with THR497, ASP503, and TYR443 of STRN3 (Figure 7B). Table S5 (Supporting Information) shows the detailed results.

To confirm the physical interaction, we induced the production of Flag‐TRAF3IP3 and HA‐STRN3 in 293T cells for mutual immunoprecipitation and verified connections between the two proteins (Figure 7C). The intense interaction between endogenous TRAF3IP3 and STRN3 in lung cancer cells was validated by co‐immunoprecipitation (Co‐IP) using A549, PC9, and H1299 cell lysates (Figure S6C, Supporting Information). Simultaneously, immunofluorescent staining indicated that TRAF3IP3 and STRN3 colocalized mainly in the cytoplasm of A549, PC9, and H1299 cells (Figure 7D). The red fluorescence curve of TRAF3IP3 closely matched the one of STRN3 (green) (Figure 7E). Additionally, we found that the expression of TRAF3IP3 was accompanied by changes in the expression of STRN3 (Figure S6D,E, Supporting Information).

The composition of TRAF3IP3 involves a transmembrane domain and two Coiled‐coil domains. Next, to examine the domain responsible for TRAF3IP3's regulation of STRN3, we prepared a wild‐type TRAF3IP3 plasmid and targeted specific domains in the TRAF3IP3 plasmids, respectively (Figure 7F). After transfected with Flag‐ TRAF3IP3 (different truncates) and HA‐STRN3 into 293T cells for 48 h, the lysates were extracted for co‐IP experiments. As shown in Figure 7G, the domain that showed the most robust interaction with STRN3 is residues 521–551(transmembrane domain), followed by residues 1–235 and 236–450. However, no interaction was observed in the domain 450–520. To further evaluate the effect of specific domains of TRAF3IP3 on the expression of STRN3, western blotting was performed to analyze the STRN3 level. Different Flag‐TRAF3IP3 plasmids or empty plasmids were transfected into A549 cells, and the results revealed that wild‐type and transmembrane domain of TRAF3IP3 overexpression markedly upregulated STRN3, as well as ER stress proteins GRP78 and ATF, and apoptosis‐related protein Bax (Figure 7H). These findings suggest that transmembrane domains are essential in STRN3 regulation. Given that TRAF3IP3 and STRN3 are proven to localize in the ER, we hypothesize that TRAF3IP3 may act as a transporter to recruit STRN3 to the endoplasmic reticulum. Immunofluorescence was performed to corroborate the impact of the transmembrane domain of TRAF3IP3 on STRN3 endogenously in LUAD cells. Indeed, after the transfection of wild‐type and transmembrane domain plasmids, an enhancement in the colocalization of STRN3 with the ER marker GRP78 was observed via confocal microscopy assays, suggesting that TRAF3IP3 recruits STRN3 to the ER in LUAD cells (Figure 7I), with the colocalization coefficient R‐value increased from 0.49 in the control group to 0.69 and 0.72 in the wild‐type group and transmembrane domain (521‐551) group, respectively. However, the colocalization interaction in the other three groups exhibited an R‐value comparable to that of the control group (Figure 7E). These results indicated that TRAF3IP3 could interact with STRN3 and promote STRN3 localization in the ER. Moreover, to better illustrate the role that the transmembrane domain plays in TRAF3IP3, control vector, wild‐type TRAF3IP3, specific transmembrane domain, and transmembrane domain‐deleted (rTM) plasmids were transfected into A549 cells with or without Thapsigargin treatment. The cell lysates were subjected to immunoblot analysis with indicated antibodies. Alterations in ER‐ and apoptosis‐related proteins (PERK, GRP78, ATF4, CHOP, Bax) showed similarities between transfecting plasmids with transmembrane domain or wild‐type plasmids (Figure 7K,L). To elucidate the affinity relationship between TRAF3IP3 and STRN3 under endoplasmic reticulum stress conditions, we co‐expressed Flag‐TRAF3IP3 and HA‐STRN3 in 293T cells. Co‐IP experiments exhibited a dramatically increased protein binding of TRAF3IP3 and STRN3 after thapsigargin treatment, while 4‐PBA reduced the interaction intensity, compared to the control cells respectively (Figure S6F, Supporting Information).

### 2.8 TRAF3IP3 Regulates ER Stress‐Related Apoptosis by Interacting with STRN3

Although previous studies have shown that STRN3 is essential for ER stress,^[^ ^28^ ^]^ it is unclear whether it is involved in cellular physiological processes like TRAF3IP3 in lung cancer, such as regulating apoptosis and prolonged ER stress. Through TCGA analysis, STRN3 was found to be up‐regulated in LUAD. However, the protein level of STRN3 was significantly lower in LUAD than in normal tissue, resembling that of TRAF3IP3 (Figure S7A, Supporting Information). In Kaplan‐Meier survival analysis, a high STRN3 level was significantly correlated with better OS and DFS (Figure S7B, Supporting Information). By western blotting analysis, we found that STRN3 presented lower expression in lung cancer cells (A549, PC9, and H1299) compared with normal bronchial epithelial cells (BEAS‐2B), with the lowest expression in A549 cells (Figure S7C, Supporting Information). We then constructed a stable expressing HA‐STRAN3 A549 cell line and STRN3‐knockdown PC9 and H1299 cell lines and confirmed the expression efficiency in cells (Figure S7D, Supporting Information). LUAD cell viability was assessed with a CCK8 assay, revealing that overexpression of STRN3 significantly reduced cell proliferation, while decreased expression of STRN3 led to an opposite effect (Figure S7E, Supporting Information). In line with the CCK8 results, the colony formation assay results also showed that overexpression of STRN3 jeopardizes the ability of the clone forming (Figure S7F, Supporting Information). Flow cytometry analysis was used to measure apoptosis levels in cell lines with altered STRN3 expression and revealed a significant increase in the proportion of apoptotic cells following the upregulation of STRN3, as shown in Figure S7G (Supporting Information). Finally, we were able to show that overexpression of STRN3 can upregulate ER stress markers such as GRP78, ATF6, PERK, and CHOP, whether under excessive ER stress or not. On the other hand, STRN3 overexpression reduced Bcl‐2 while increasing Bax and cleaved‐caspase 3, indicating increased cell apoptosis. Conversely, we also confirmed the role of STRN3 in regulating ER stress in PC9 and H1299 knockdown cells (Figure S7H–J, Supporting Information).

**Figure 8:**
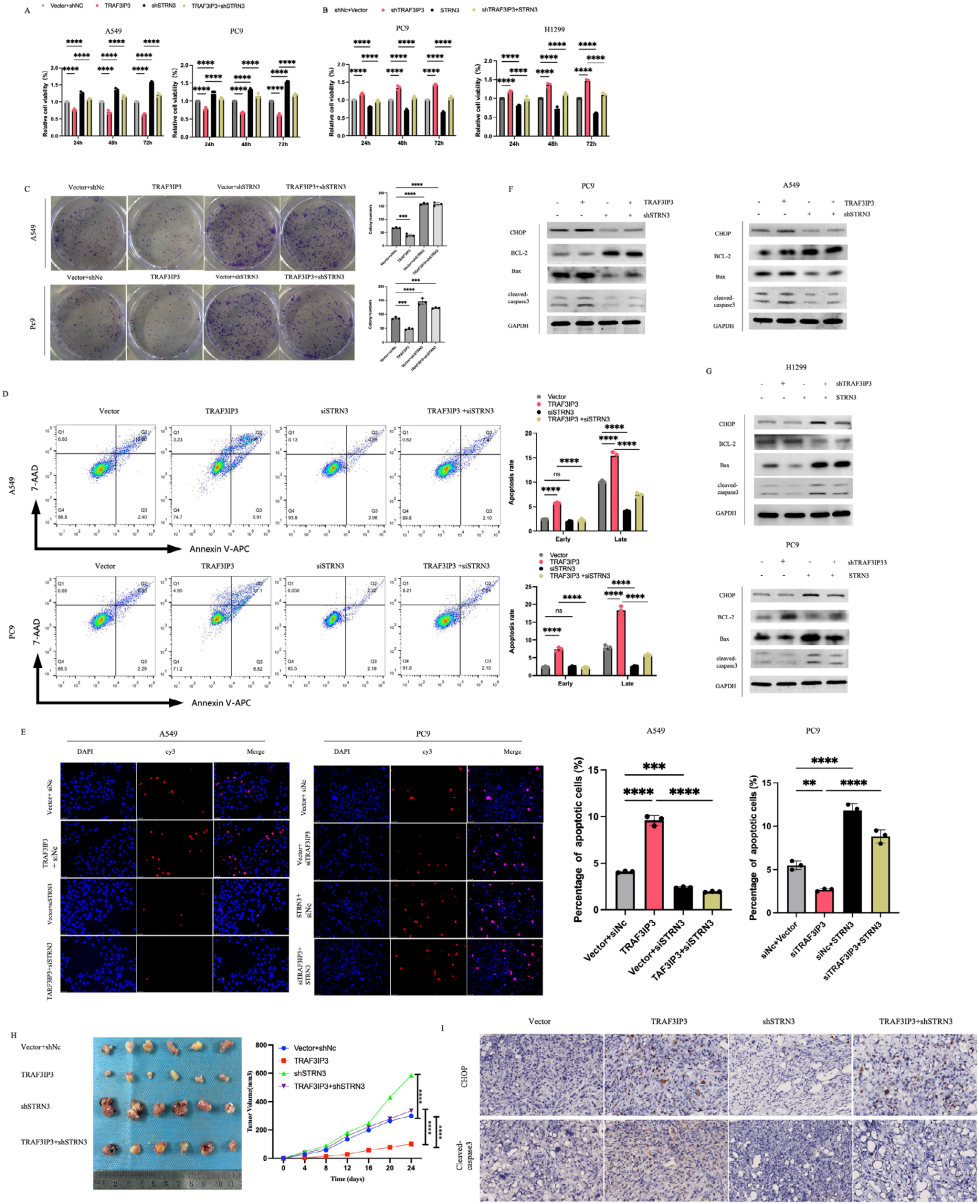
TRAF3IP3 regulates ER stress‐related apoptosis by interacting with STRN3. A, C) The effect of vector+shNc, Over‐TRAF3IP3+shNc, vector+shSTRN3, and over‐TRAF3IP3+shSTRN3 on cell viability (A) and colony formation capability (C) in A549 and PC9 cells was assessed using the CCK8 assay and colony formation assay. B) The effect of vector+shNc, vector+shTRAF3IP3, over‐STRN3+shnc, and over‐STRN3+shTRAF3IP3 on cell viability in PC9 and H1299 cells was assessed using the CCK8 assay. D) Flow cytometry was performed to determine the effect of vector+shNc, over‐TRAF3IP3+shNc, vector+shSTRN3, and over‐TRAF3IP3+shSTRN3 cells on cell apoptosis of A549 and PC9 cells. E) TUNEL assay was performed to detect the apoptotic changes of A549 and PC9 by transfecting indicated vectors or siRNAs. F, G) The expression patterns of apoptosis‐related proteins in A549, PC9 and H1299 cells were evaluated by Western blotting. H) Nude mice were injected subcutaneously with 2×10^6^ A549 cells expressing empty vector, TRAF3IP3 overexpression, shSTRN3 or TRAF3IP3 overexpression+shSTRN3. Visual representations of tumor growth curves from the indicated cell lines were showcased. I) Images showing immunohistochemical analysis of xenografts tumor tissues using specified antibodies in each group. The immunohistochemical staining process was repeated three times independently. Calculation of positive cells in the staining was performed. A, B, C, D, E, H n = 3, *t*‐test and one‐way ANOVA, ns: *p* ≥ 0.05, **p < 0.01, ***p < 0.001, ****p < 0.0001.

To explore whether TRAF3IP3 serves its biological function through STRN3, rescue experiments were designed using STRN3 mimics and ShSTRN3 cell lines. As evidenced by functional assays including CCK8 assay (**Figure** **8**A,B), colony formation assay (Figure 8C; Figure S8A, Supporting Information), flow cytometry assay (Figure 8D; Figure S8B, Supporting Information), and TUNEL assay (Figure 8E), STRN3 deletion reverses the inhibitory effect of TRAF3IP3 overexpression on proliferation and colony‐forming ability in A549 and PC9 cells, which was also confirmed in reverse experiments in TRAF3IP3 depletion cell lines. Besides, TRAF3IP3‐induced alterations in ER‐stress proteins (GRP78, ATF6, p‐PERK) and apoptosis‐associated proteins (Bax, cleaved‐caspase 3, Bcl‐2, and CHOP) were counteracted by STRN3 (Figure 8F,G, Figure S8C, D, Supporting Information). Consistent with this, depletion of STRN3 significantly compromised TRAF3IP3‐mediated inhibition of subcutaneous xenograft tumors in vivo assays (Figure 8H; Figure S8E, Supporting Information), as well as CHOP and Cleaved‐caspase3 expression (Figure 8I; Figure S8F, Supporting Information). Collectively, these results suggested that TRAF3IP3 suppressed LUAD proliferation via interacting with STRN3 in vitro and in vivo (**Table** **1**).

**Table 1:** Upregulation of TRAF3IP3 correlates with Lung adenocarcinoma progression.

	TRAF3IP3mRNA				*P* Value (Fisher's Test)
Characteristic	Low expression	High expression	n	Increased [%]	
**Age**, years					
<60	13	21	34	61.8%	0.106
≥60	17	12	29	41.4%	
**Sex**					
Female	14	15	29	51.7%	0.923
Male	16	18	34	52.9%%	
**Smoking history**					
Yes	20	15	35	48.4%	0.260
No	12	16	28	57.1%	
**Differentiation**					0.017
Low	11	4	15	26.7%	
Moderate or high	19	29	48	60.4%	
**Lymph node metastasis**					
None	12	23	35	65.7%	0.018
N1‐N3	18	10	28	35.7%	
**Location**					
Peripheral	19	15	34	48.4%	
Central	13	16	29	20.7%	
**Pulmonary lobe**					
Left	10	20	30	66.7%	0.03
Right	20	13	33	39.4%	
**Tumor stage**					
I	10	22	32	68.8%	0.029
II	11	7	18	38.9%%	
III	9	14	23	60.9%%	
T stage					
T1	4	22	26	84.6%%	<0.001
T2	22	11	33	33.3%	
T3	4	0	4	0%	
**Distant metastasis**					
M0	31	32	63	50.8%	‐
M1‐M3	0	0	0		

Fisher's exact test was used to test the association between the categorical variables;

^*^Statistically significant, *p* < 0.05.

### 2.9 IKZF1 Regulates TRAF3IP3 Transcription to Suppress LUAD Proliferation

To further explore the expression and activation mechanism of TRAF3IP3, we used online database UCSC (https://genome.ucsc.edu/) to predict transcription factors that may bind to TRAF3IP3. According to prediction results on the UCSC (Figure S9A, B, Supporting Information), several transcription factors could potentially bind to TRAF3IP3 and probably regulate its expression, among which we selected three transcription factors that are most likely to bind to the promoter region of TRAF3IP3—RUNX3, IKZF1, and MEF2B. The relationship between TRAF3IP3 and the expression of the above three potential transcription factors in lung adenocarcinoma were analyzed using the TIMER online database, and the results showed that there is significant positive correlation between TRAF3IP3 and the expression of all the above transcription factors in lung adenocarcinoma, with r = 0.769, 0.893, and 0.464 respectively (Figure S9C, Supporting Information). Furthermore, we analyzed the expression of RUNX3, IKZF1, and MEF2B in lung adenocarcinoma and normal lung tissues using TCGA data, and the analysis indicated that the three transcription factors were all downregulated in lung adenocarcinoma tissues (Figure S9D, Supporting Information). We therefore speculated that the expression of TRAF3IP3 might be regulated by these transcription factors and it might be expressed at low levels in LUAD cells. As IKZF1 showed the highest correlation with TRAF3IP3, we chose IKZF1 to explore whether it can bind to TRAF3IP3 and regulate its transcription.

Depleting or overexpressing IKZF1 reduced or increased TRAF3IP3 at the mRNA level, suggesting that IKZF1 may regulate TRAF3IP3 expression (Figure S9E, Supporting Information). To test whether IKZF1 binds to the TRAF3IP3 promoter region, the binding sequences of IKZF1 to the TRAF3IP3 promoter region were searched using the UCSC database and primers were designed segmentally according to the sequences (Figure S9F, Supporting Information). ChIP was then performed using the IKZF1 antibody, and the extracted DNA was detected by qRT‐PCR using the above primers, followed by agarose gel electrophoresis. The results indicated that IKZF1 binds significantly to sites BD of the TRAF3IP3 promoter region (Figure S9G, Supporting Information). In addition, to determine whether TRAF3IP3 expression could rescue the effects of IKZF1 on LUAD cell proliferation, siRNAs (IKZF1 or TRAF3IP3) and overexpression plasmids (IKZF1 or TRAF3IP3) were co‐transfected into PC9 and A549 cells. CCK‐8 assay showed that IKZF1 could inhibit LUAD cell proliferation, while TRAF3IP3 silencing could partially reverse IKZF1's inhibitory effect on LUAD cell proliferation (Figure S9H, Supporting Information). Based on these results, TRAF3IP3 can be regulated by transcription factors which exhibit a low level in LUAD cell lines.

## 3 Discussion

Cancer is the leading cause of death worldwide and remains a major obstacle to increasing life expectancy in all countries, among which lung cancer has the highest incidence and mortality rate.^[^ ^29^ ^]^ The molecular mechanisms involved in developing lung cancer and identifying novel markers or therapeutic targets have become a hot area for current researchers. TRAF3IP3 was previously reported as an adapter protein that plays essential roles in innate and adaptive immunity and could also respond to viral infection,^[^ ^5^, ^30^ ^]^ indicating its potential adjuvant role in hosts in cancer therapy. However, its role in lung cancer remains unknown. Through bioinformatics analysis using the biological database (TCGA, UALCAN), we found that TRAF3IP3 was highly expressed in several tumor tissues. In contrast, it was downregulated only in lung cancer, especially at the protein level. The proteomic analysis revealed that TRAF3IP3 was significantly correlated with ER stress non‐folded protein response and autophagy, indicating that TRAF3IP3 may be a novel ER stress‐related protein or have a function across autophagy. In the present study, we report that TRAF3IP3 can trigger sustained and potent ER stress in lung adenocarcinoma cells, leading to the decline of cell growth and enhancement of cell apoptosis. ER stress can emerge as a double‐edged sword that could either maintain cellular homeostasis or contribute to cell fate termination. Notably, we showed that TRAF3IP3 induces ER stress‐mediated apoptosis by interacting and transferring an ER protein STRN3 in the ER lumen to stimulate the PERK/ATF4/CHOP pathway, one of the three pathways in UPR activation. Given that autophagy could be activated by the UPR pathway to restore cellular homeostasis, we also assessed and testified the correlation between TRAF3IP3‐induced ER stress and autophagy in this work. Therefore, for the first time, we decipher the specific mechanism of TRAF3IP3 involved in cell suppression in LUAD patients.

According to online database analysis, we found that the TRAF3IP3 protein level was much lower only in LUAD and LUSC tissues. Meanwhile, the K‐M results revealed its clinical significance only within LUAD patients. Consistently, the expression level of TRAF3IP3 was dramatically downregulated in patients with primary lung adenocarcinoma. A few studies have investigated the functional role in cancers. For instance, TRAF3IP3 could facilitate glioma cell proliferation, migration, and invasion, at least partly by activating the ERK signaling pathway.^[^ ^5^ ^]^ In addition, TRAF3IP3‐shRNA in melanoma tumors showed an increase in apoptosis and a decrease in microvascular density, which supported a proangiogenic and a direct protumoral role in melanoma.^[^ ^31^ ^]^ However, the exact role of TRAF3IP3 in tumors depends on the cancer type or context. Herein, in LUAD cancer cells, A549 and PC9 TRAF3IP3‐overexpression cell lines displayed decreased cancer proliferation and increased apoptosis without cytotoxic effects on cancer cell migration and invasion ability. Proteomic analysis suggests that the ER stress pathway may be involved in TRAF3IP3‐mediated tumor suppression and autophagy. The use of various inhibitors of the cell death pathway in TRAF3IP3‐overexpressing cell lines further increased our interest in these two processes.

The ER is an organelle where proteins synthesize, fold, and mature. Conditions such as hypoxia, low glucose, cell starvation, or gene mutations could cause damage to ER function, thus leading to ER stress.^[^ ^12^ ^]^ Cells use the three UPR sensors to maintain homeostatic balance through phosphorylates eIF2a, ER‐associated degradation (ERAD), and IRE1‐dependent decay (RIDD). In cases of severe ER stress, PERK can promote apoptotic cell death by stimulating ATF4, which subsequently activates the transcription factor CHOP; CHOP then triggers caspase activation by regulating genes such as Bim and DR5.^[^ ^32^ ^]^ In addition, PERK can promote IRE1 degradation by dephosphorylating IRE1 via the phosphatase RPAP2, which in turn inhibits the activity of IRE1 in the face of unmitigated ER stress, thereby aborting the cytoprotective adaptation and promoting DR5‐mediated apoptosis.^[^ ^24b^ ^]^ In the current study, we found that TRAF3IP3 could lead to the upregulation of ER stress‐associated proteins, including PERK, ATF6, IRE1, and GRP78, and it could further increase these protein levels under thapsigargin‐induced ER stress, indicating its role in regulating ER stress. We also showed that elevated TRAF3IP3 induces LUAD cell apoptosis, and a higher apoptosis level was observed under thapsigargin‐induced ER stress. To figure out the mechanism by which TRAF3IP3 triggered apoptosis, siRNAs targeting PERK and ATF4 were applied. Flow cytometry analysis demonstrated that the apoptotic rate reversed majorly in TRAF3IP3‐overexpression cells with depletion of PERK or ATF4, and the CHOP level and proapoptotic protein levels such as Bax and cleaved‐caspase3 were decreased, accompanied by increased level of the antiapoptotic protein Bcl‐2. TRAF3IP3 has been shown to localize to the Golgi, lysosomes, and plasma membrane in previous studies.^[^ ^33^ ^]^ For the first time, we found that TRAF3IP3 localized to the ER in LUAD cells, and the level of TRAF3IP3 and GRP78 can both be upregulated under thapsigargin‐induced ER stress. Autophagy is also a conserved process that can be regulated by ER stress, degrading the proteins of the ER within lysosomes or vacuoles.^[^ ^17^, ^34^ ^]^ It was reported to be activated through the activation of ATF4, which leads to the upregulation of autophagy genes LC3 and Atg12, or stimulate ATF6 to activate the Rheb‐mTOR pathway to promote the autophagy initiation, or via the IRE1 mediated autophagic process.^[^ ^13^, ^35^ ^]^ ER stress‐mediated autophagy is critical to alleviate ER stress and prevent cancer cell death by removing ubiquitinated misfolded/unfolded proteins, thus promoting tumor survival.^[^ ^19b^ ^]^ The western blotting results showed that the autophagy marker LC3B was consequently reduced under 4‐PBA or slicing ATF4, implying the autophagy induced by TRAF3IP3‐mediated ER stress. This was visually verified through the use of a transmission electron microscope. The selective clearance of ER subdomains is involved in controlling ER size and activity during ER stress, restoring ER homeostasis after ER stress is resolved, and removing parts of the ER where aberrant and potentially cytotoxic material has segregated.^[^ ^17^, ^36^ ^]^ We examined whether the autophagic flux induced by TRAF3IP3 could result in cancer cell death or have a protective effect. The role of autophagy in cancer cells is highly cell‐type and context‐dependent and is known to have either an oncogenic or tumor suppressor role in cancers. In our study, we examined the linkage between autophagy and cell death in TRAF3IP3‐overexpressing LUAD cells and found that cancer cell death was promoted in cells slicing ATG7 or treated with autophagy inhibitor in those cells, indicating that autophagy contributes to the promotion of survival of TRAF3IP3‐overexpressing human lung cancer cells. Thus, TRAF3IP3 may be a promising prognostic biomarker related to autophagy.

To explore the specific mechanism of TRAF3IP3 in such signaling in LUAD cells, we performed mass spectrometry and discovered a novel substrate, STRN3, that can interact with TRAF3IP3 strongly. STRN3 is one of the B’’’ subunits members of heterotrimeric PP2A holoenzyme's B subunit and was well identified as central scaffolds of a class of multi‐subunit complexes called striatin‐interacting phosphatases and kinases (STRIPAK).^[^ ^37^ ^]^ The (STRIPAK) complex is a set of evolutionarily conserved multiprotein scaffolded assemblies that can regulate cellular processes, including vesicular trafficking, hippocampal signaling, autophagy, cell differentiation, metabolism, and programmed cell death, among which both TRAF3IP3 and STRN3 are involved.^[^ ^38^ ^]^ Studies have reported that high STRN3 level was closely linked to YAP hyperactivity and GC prognosis and was associated with a lower differentiation stage in human GC, indicating that STRN3 may be a potential differentiation status indicator.^[^ ^39^ ^]^ Other studies reported that the presence of STRN3 alongside the antioxidant DJ‐1 protein safeguards nerve and cancer cells from oxidative tension. They also showed a downregulation of STRN3 in lung cancer A549 and H1299 cell lines, and the expression of STRN3 differs between cancer types.^[^ ^40^ ^]^ In addition, Bisoyi P et al. first investigated the regulation of STRN3 in response to ER stress. They found that STRN3 is a component of the endoplasmic reticulum homeostasis regulatory system and that reduced expression of STRN3 leads to apoptosis in mouse fibroblasts in response to enhanced ER stress.^[^ ^28^ ^]^ However, the exact role of STNR3 in lung cancer is still unknown. In the current study, we validated the anti‐tumor properties of STRN3 in lung adenocarcinoma through a series of experiments on cell functions. Furthermore, our results first established the substantive role of STRN3 in regulating ER stress in lung cancer cells and found that sustained high expression levels of STRN3 could facilitate the intensity of ER stress in lung cancer cells, which was discrepant with the effects in mice fibroblast cells. Based on these, we asked if TRAF3IP3 triggers ER stress and ‐associated apoptosis by interacting with STRN3. In light of this, a set of rescue procedures was performed, confirming that TRAF3IP3 restrains the cell proliferation rate and enhances the apoptosis of LUAD in an STRN3‐dependent manner.

To our best knowledge, TRAF3IP3 was initially identified as a TRAF3 interacting protein, and it was proved to be indispensable in the maturation and stability of regulatory T cells through ERK signaling^[^ ^5^ ^]^ and mTORC1signaling,^[^ ^33^ ^]^ and the impact of TRAF3IP3 also differs upon virus infection.^[^ ^41^ ^]^ Therefore, TRAF3IP3 seems to serve various roles, which vary according to the cell type. It consists of two coiled‐coil domains and a transmembrane domain. Exogenous and endogenous co‐immunoprecipitation were performed to understand the exact interactions between TRAF3IP3 and STRN3. Results showed that TRAF3IP3 had a robust interaction with STRN3. Studies have demonstrated that the presence of the transmembrane region in TRAF3IP3 is crucial for its localization within lysosomes and its function in orchestrating the recruitment of PP2A to hinder mTORC1 signaling in T reg cells, prompting us to speculate that the TRAF3IP3 transmembrane domain is responsible for its impact on ER stress and apoptosis. Following this, experiments were conducted to map the domains involved using STRN3 and deletion truncation of TRAF3IP3. STRN3 was confirmed to localize at the ER and induce ER stress based on our study. We consequently obtained strong evidence that the TRAF3IP3 could facilitate the ER recruitment of STRN3 in LUAD cells. Notably, the ability of TRAF3IP3 to mediate the recruitment of STRN3 to the ER depended on its transmembrane domain. Thus, TRAF3IP3 enables STRN3 to accelerate ER stress and apoptosis by facilitating the localization of STRNS to the ER. To better understand the mechanism of action of TRAF3IP3, we also sought to identify the potential transcription factors that could regulate its expression. According to the UCSC database, RUNX3, IKZF1and MEF2B were proposed to be most likely to bind to the promoter region of TRAF3IP3 and exhibited a significant positive correlation in LUAD. We preliminary proved that IKZF1 binds to the promoter region of TRAF3IP3 and activates the transcription of TRAF3IP3.

To summarize, the present study first demonstrated that TRAF3IP3 localizes to the ER in LUAD cells and inhibits tumor cell proliferation in vitro and in vivo. Specifically, TRAF3IP3 can interact with STRN3 intently and transfer it to ER to promote ER stress‐related apoptosis. Notably, we demonstrated that TRAF3IP3 simultaneously promotes cytoprotective autophagy alongside ER stress, drawing the potential of combined treatment with pharmacological autophagy inhibitors. Moreover, based on its essential role in T cells, there is a temptation for speculation that TRAF3IP3 could represent a revolutionary therapeutic in LUAD target or immunotherapy.

## 4 Experimental Section

### LUAD Patient Samples and Cell Culture

Primary LUAD specimens and corresponding adjacent non‐tumor normal tissues were obtained from patients who underwent radical pulmonary resection at the First Affiliated Hospital of Xi'an Jiaotong University between 2015 and 2020. All the tumors or normal tissues utilized in the study were frozen in liquid nitrogen instantly after resection and stored at −80 °C. The study was approved by the Ethics Committee of the First Affiliated Hospital of Xi'an Jiaotong University (No. 2021–560) and informed consent was obtained from all LUAD patients. The clinicopathological characteristics of these 126 patients are provided in Table S1 (Supporting Information).

LUAD cell lines (PC9, A549 and H1299), bronchial epithelioid cells (BEAS‐2B), and Human embryonic kidney (HEK) 293T cells were all purchased from the American Type Culture Collection (ATCC, Manassas, VA, USA). Cell lines were incubated in the appropriate growth media (PC9 and H1299: RPMI‐1640 (Gibco, USA); A549: DMEM (Gibco, USA)) containing 10% FBS (Gibco) at 37 °C in a humidified incubator with 5% CO^2^. All cell lines were analyzed using short tandem repeat (STR) for authentication and regularly detected for mycoplasma contamination.

For the ER stress drug experiment, stably expressed cell lines were seeded in 6‐well plates until the cells grew to the confluence of ≈70–80%. UPR‐mediated apoptosis can be triggered in cancer cells by ER stress inducers like thapsigargin and tunicamycin.^[^ ^42^ ^]^ Then, the ER stress inducer Thapsigargin and inhibitor 4‐PBA were used to stimulate or rescue ER stress conditions, with a concentration of 1 µM and 1 mM, respectively, which refer to previously reported. To induce autophagy, cells were starved by incubating in HBSS medium (GIBCO) or medium without FBS. 20 µM chloroquine (CQ, Sigma–Aldrich) was used for 8 or 12 h to inhibit lysosomal degradation.

### Annexin V–APC/7‐AAD Apoptosis and TUNEL Assay

A549, PC9, and H1299 cells were seeded in 6‐well plates and cultured overnight. A549 and PC9 cells were used to construct TRAF3IP3‐overexpressing cell lines, and PC9 and H1299 were used to construct TRAF3IP3‐knockdown cell lines. After 48 hours of indicated treatment, the culture medium was collected and cells were resuspended and stained with Annexin V‐APC and 7‐AAD using Annexin V‐APC/7‐AAD Apoptosis Kit (Elabscience, E‐CK‐A218) according to the manufacturer's protocol. A flow cytometer (Bio‐Rad ZE5 Analyzer) was used to measure the resulting fluorescence within one hour.

TUNEL staining (TUNEL kit, Beyotime) was used to analyze cell apoptosis. It was performed according to the indicated protocols. To analyze the data, we collected more than eight image fields from each well and consolidated the counts from all images within a single well to serve as a replicate for statistical analysis (n = 3 wells of each group).

### Immunohistochemistry

The immunohistochemistry procedure was conducted following established protocols,^[^ ^43^ ^]^ using antibodies targeting TRAF3IP3 (Proteintech, 1:200), Ki67 (Proteintech, 1:2000), CHOP (Proteintech, 1:200), STRN3 (Abclonal, 1:100) and Cleaved caspase‐3 (CST, 1:400). Primary human LUAD and normal tissues, as well as the xenograft tissue of nude mice, was taken for immunohistochemical staining to assess the indicated protein expression.

### Western Blot Analysis

Western blot analyses were conducted according to the indicated protocols as previously described. Tissue (from LUAD sample or mice xenograft model) and cellular protein were extracted using RIPA Lysis Buffer (Thermo Scientific, USA). Protein concentrations of supernatants were measured with the BCA protein detection kit (Beyotime, China). They were separated using 10% SDS‐PAGE and transferred to the PVDF (immobilon Millipore, Sigma, USA) membrane with the wet‐transfer method, followed by 5% skimmed milk powder for one hour at room temperature. Membranes were incubated with primary antibodies at 4 °C overnight. The antibodies used are shown in Table S2 (Supporting Information).

### Cell Functional Assays

To evaluate the in vitro functional roles of TRAF3IP3, the CCK8 proliferation assay, colony formation assay, migration assay, and wound healing assay were performed. For the CCK8 assay, cells in the logarithmic growth phase were seeded in 96‐well plates at the appropriate density (≈2000 cells per well). Six duplicate wells and a negative control were prepared for each group. Each well was added with 10 µL of the CCK8 (TargetMol, USA) substrate. The 96‐well plates were then incubated at 37 °C for 1.5 h, and the OD values were examined at a wavelength of 490 nm with a microplate reader. The proliferation curve was generated by drawing the absorbance change curve over time. For colony formation assay, cells were seeded in a 6‐well plate with a concentration of ≈1000 cells per well. After being cultured in a refreshed medium for two weeks, surviving colonies (more than 50 cells per colony) were assessed through staining with 0.05% crystal violet (n = 3 wells of each group). The migration assay was performed using an 8.0‐µm 24‐Transwell permeable support (Corning, USA). Cells (PC9, A549, and H1299:2×104) were seeded in the upper chamber with 200 µL serum‐free medium, and 600 µL medium containing 10% FBS was applied to the lower chamber. After incubating for migration at 37 °C for 24 or 48 h, a cotton swab was used to remove the cells in the upper filter of the chamber, and the migration cells were fixed and stained with 4% paraformaldehyde solution and 0.1% crystal violet. The migrated cells were counted at five fields under a Nikon inverted microscope. In the wound healing assay, a 10 µL pipette tip was used to scratch the confluent monolayer of cells. Cell migration into the wound was then detected by microscopy after 24 or 48 h.

### Establishment of Stable Cell Lines, Plasmids, and siRNA Knockdown

Lentiviral vectors were used to stably overexpress TRAF3IP3 and STRN3, or knockdown TRAF3IP3 and STRN3 in PC9, A549 and H1299 cells. ShRNAs targeting TRAF3IP3 and STRN3 were cloned into the pSuper retroviral vectors with different antibiotic resistance genes. Further, the viral particles were infected into PC9, A549, and H1299 cells, and stably integrated cells were selected with 5 µg mL^−1^ puromycin for 1–2 weeks. Cells were grown in media with 2 µg mL^−1^ puromycin at 37 °C in a humid incubator with 5% CO^2^. The FLAG‐TRAF3IP3 and HA‐STRN3 plasmid constructs were cloned into a pCM‐3xFlag‐BGH‐Neo or a pCMV‐MCS‐3xFlag‐WPRE‐NEO vector (MIAOLING Biology, China). qRT‐PCR and western blots were performed to confirm all stable cell lines before further investigation.

For transient transfection, small interfering RNAs (siRNAs) targeting TRAF3IP3, STRN3, PERK, and ATF4, as well as negative controls, were directly synthesized by HanBiotechnology, China. siRNA transfection was conducted using the Lipo8000 transfection reagent (Beyotime) according to the manufacturer's instructions. Cell samples were collected 48 hours after transfection for RNA and protein analyses.

### Transmission Electron Microscopy

Transmission electron microscopy (TEM) was applied to analyze the ultrastructural changes of the endoplasmic reticulum in cells. After the indicated treatment, PC9 and A549 cells were washed with PBS and collected in a 1.5 mL EP tube. Cells were pre‐fixed with 2.5% glutaraldehyde + 4% paraformaldehyde at 4 °C for 2 h, washed with 0.1 M phosphate buffer, and then post‐fixed with 1% osmium acid at 4 °C for 2 h before being dehydrated with gradient concentrations of acetone. The samples were subsequently embedded, polymerized, and sectioned. Ultrathin sections were stained with uranyl acetate and lead citrate for 15 min and examined by a HITACHI H‐7650 transmission electron microscope.

### RNA Isolation and Quantitative Real‐Time PCR

The total RNA of indicated cells and tissues was extracted using a Fast 500 kit (Fastagen, China) according to the manufacturer's instructions. The synthesis of cDNAs from mRNAs was carried out with Hifair II 1st Strand cDNA Synthesis SuperMix for qPCR (Yeasen, Shanghai, China) following the standard protocols. By using Hieff qPCR SYBR Green Master Mix (No Rox) (Yeasen, Shanghai, China), qRT‐PCR was conducted on the CFX96 Real‐time PCR detection system (Bio‐Rad). The comparative 2−ΔΔCt method was employed to calculate the relative expression of the experimental groups, with GAPDH serving as the internal control. All the primers used in the study are shown in Table S2 (Supporting Information).

### Fluorescence Microscopy

For immunofluorescence staining, cells are cultured in sterile confocal dishes after the indicated treatment and washed twice with PBS, followed by fixation with 4% paraformaldehyde for 20 min. Thereafter, the cells were permeabilized in 0.2% Triton X‐100 (Sigma‐Aldrich, China) at 4 °C for 10 min and blocked in PBS‐B (4% BSA) for 1 h. The cells were then fluorescently double‐stained with primary antibodies against TRAF3IP3(Santa, sc‐398895), STRN3 (Abclonal, A6756) or GRP78(Proteintech, 66574‐1‐lg) at 4 °C overnight. Fluorescence‐conjugated secondary antibodies were subsequently added and incubated at room temperature in the dark for 2 h. Nuclei are stained with DAPI. Visualizations of cells and image capture were carried out using a confocal microscope (Leica, Germany).

For autophagic flux examination, A549 (or PC9) cells overexpressing TRAF3IP3 were seeded in the confocal dish and transfected with appropriate HBAD‐merry‐EGFP tagged LC3B for 24 h, with or without CQ (20 µM) treatment. The confocal dishes were directly taken to take pictures. All the images were captured using a confocal microscope (Leica, Germany) with a 40‐oil objective. Autophagosomes were identified as RFP + GFP + (yellow dot), whereas mature autolysosome organelles were identified as RFP + GFP – (red‐only dot).

### Molecular Docking

PDB files of the 3D structures of human TRAF3IP3 and STRN3 were obtained from the Protein Data Bank (PDB, www.rcsb.org) database. Pymol (version 2.3.0) (https://pymol.oT) was used to separate primitive ligands and protein structures, dehydrate, and remove organic matter. The prepare module in Discovery Studio software was applied for protein preparation, e.g., hydrogenation and protonation production. HDOCK was used to analyze protein‐to‐protein docking and its binding activities in different conformations and amino acid residues with interaction distances up to 5A. During the docking process, TRAF3IP3 was considered the docking acceptor, and STRN3 was considered the docking ligand. 100 poses were generated from the docking results, and the TOP10 conformations were extracted using the Creapl script and visualized by Pymol. Finally, the TOP1 was selected for visualization and analysis. Protein interaction interfaces were analyzed via the analysis interface module in the Discovery studio.

### Co‐Immunoprecipitation (Co‐IP)

For immunoprecipitation, 293T cells were transfected with FLAG‐TRAF3IP3 and HA‐STRN3 plasmids using jetPRIME (Polyplus, USA). After 48 h of incubation, cells were collected and lysed with RIPA buffer at 4 °C for 1 h to obtain a better lysate. Then, the cell lysates were incubated with the indicated antibodies (anti‐Flag, Proteintech, China; anti‐HA, Proteintech, China) at 4 °C overnight. After that, Dynabeads protein G (Invitrogen, USA) was added to the antigen‐antibody complex and incubated at 4 °C for 4 h before being washed. The resultant beads were washed three times with PBS before further Western blot analysis.

### Liquid Chromatography‐Mass Spectrometry

PC9 cells were transfected with Flag‐tagged TRAF3IP3 or vector control plasmids to construct stabilized overexpressed cell lines. Label‐free quantitative proteomics analysis was performed by Aksomics, biotech (Shanghai, China) to detect differentially expressed proteins. The two groups of PC9 cells were cultured and collected. Peptides underwent examination using a Q Exactive mass spectrometer (Thermo Fisher) in conjunction with the Nano‐UPLC Liquid Chromatography system EASY‐nLC1200 (Thermo Fisher). The full scan and subsequent MS/MS analysis were done with resolutions of 70 000 for MS1 and 17 500 for MS2 at M/Z 200. The maximum ion injection time (Max IT) was 50 ms for MS1 and 45 ms for MS2, with automatic gain control (AGC) set to 3E+6 and 1E+5, respectively. The normalized collision energy (NCE) is 28%, the isolation window is 2 m/z, and the dynamic exclusion time is 40 s. Raw files were processed using MaxQuant (2.0.1.0). The protein database was obtained from the UniProt database (uniprot‐proteome‐human‐2021.2. fasta). Protein sequences and their reverse decoy sequences were also used for MaxQuant searches. Proteins with expression fold difference ratio A/B ≥1.5, p‐value≤0.05, unique peptide≥2 were defined as significantly different and subjected to subsequent GO, heatmap, and volcano plot analysis and presentation.

For interacting protein qualitative analysis, immunoprecipitation was performed. The IP protein was obtained using an anti‐Flag antibody, and the two groups of proteins were subjected to electrophoresis in SDS‐PAGE gels. The gel's proteins were broken down into peptides and removed, followed by the detection of these peptides using liquid chromatography and mass spectrometry, and ultimately, the identification of the peptides' specific details through data analysis. The indicated scores were used to screen for proteins and peptides that could specifically bind to TRAF3IP3.

### Tumor Engraft Model Establishment

The male BALB/c nude mice were all purchased from the Experimental Animal Center of Xi'an Jiaotong University and were 4–5 weeks old. The nude mice were housed in a specific‐pathogen‐free environment with the temperature kept at 23 °C ± 2 °C, humidity 40–60%, and light/dark cycles lasted 12 hours, as well as unlimited access to food and water. Animal experiments were approved by the Medical Ethics Committee of Xi'an Jiaotong University (No. XJTUAE20‐2507).

The PC9 cell line with overexpression of TRAF3IP3 and A549 cell line with TRAF3IP3 knockdown (1×10^6^ cells suspended in 100 µL PBS), as well as the control cell line, were injected subcutaneously into both sides of the axilla of the nude mice (*n*  =  4 mice per group). After about 1‐week post‐injection, subcutaneous tumors began to form, and the tumor volume was monitored every 3 days with vernier calipers. The volume was measured using the formula V = 0.5 × W^2^ × L (V, volume; L, length; W, width). In rescue animal experiments that followed, the A549 cell line stably expressed shNc+vector, TRAF3IP3, shSTRN3, and Vector +shSTRN3 (1×10^6^ cells suspended in 100 µL PBS) were injected unilaterally into the axilla of nude mice. Six mice were involved in each group. After being raised in specific conditions for about 4 weeks, the nude mice were sacrificed, and the xenograft tumors were excised and weighed, followed by fixing for further Western blot or immunohistochemistry analysis.

### Bioinformatical Analysis

An analysis of TRAF3IP3 expression in human LUAD and LUSC was conducted using TCGA datasets (GEPIA). The predictive value of TRAF3IP3 in lung cancer was obtained from Kaplan‒Meier Plotter database. The expression profile of the TRAF3IP3 protein was analyzed in the UALCAN online database. The online databases used in the study are listed in Table S3 (Supporting Information).

### Statistical Analysis

GraphPad Prism 9.0 (GraphPad Software, La Jolla, CA) was applied to statistical analysis for indicated data. The results were presented as the mean ± standard deviation (SD) from a minimum of three separate biological experiments. The comparison between two independent groups was conducted using the two‐tailed student's t‐test. Multi‐group comparisons were analyzed by one‐way ANOVA followed by Dunnett's test for specific subgroup analyses. A p‐value of less than 0.05 was considered statistically significant.

### Ethics Statement

The Ethics Committee of the First Affiliated Hospital of Xi'an Jiaotong University approved all experiments involving human samples, and informed consent was obtained from all LUAD patients. Animal experiments were approved by the Medical Ethics Committee of Xi'an Jiaotong University. Both animal care and experimentation followed all ethical guidelines for animal research.

## Conflict of Interest

The authors declare no conflict of interest.

## Author Contributions

G.Z. and J.Q. contributed equally to this work. G.Z., B.Z., C.H., and H.R. designed and analyzed the research; G.Z. wrote the manuscript; B.Z., C.H., and X.Y. helped with the validation and modification of the manuscript. G.Z. and J.Q. performed the revised data experiments. F. L., H.T., and S.D. contributed to the data analysis; R.W., Y.F., and J.W. collected the clinical samples. All authors contributed to the article and approved the submitted version.

## Supporting information



Supporting Information

## Data Availability

The data generated in this study are available upon request from the corresponding author.

## References

[1] F. Kamangar , G. M. Dores , W. F. Anderson , J. Clin. Oncol. 2023, 41, 5209. 38016281 10.1200/JCO.23.00864

[2] W. D. Travis , E. Brambilla , G. J. Riely , J. Clin. Oncol. 2013, 31, 992. 23401443 10.1200/JCO.2012.46.9270

[3] a) L. The , Lancet 2024, 403, 2663; 38908864 10.1016/S0140-6736(24)01299-6

[4] D. A. B. H. , E. D. C. S. , S. A. A. , E. G. C. D. , C. B. C. D. G. , FEBS Lett. 2003, 553, 403.14572659

[5] Q. Zou , J. Jin , Y. Xiao , H. Hu , X. Zhou , Z. Jie , X. Xie , J. Y. H. Li , X. Cheng , S.‐C. Sun , J. Exp. Med. 2015, 212, 1323. 26195727 10.1084/jem.20150110 PMC4516800

[6] a) Y. Xiaoyan , T. Xiao‐Lu , W. Feixiang , Z. Yuhan , Q. Guojun , Z. Yan , H. Zhilin , W. Zhongqiu , C. Yuzhou , C. Lei , J. Exp. Med. 2018, 215, 2463.30115741

[7] S. Nasim , O. D. Iancu , W. Guanming , J. E. Jacobs , S. K. Mcweeney , Front. Genet. 2018, 9, 183. 29910823 10.3389/fgene.2018.00183 PMC5992410

[8] a) R. Bhati , C. Patterson , C. A. Livasy , C. Fan , D. Ketelsen , Z. Hu , E. Reynolds , C. Tanner , D. T. Moore , F. Gabrielli , Am. J. Pathol. 2008, 172, 1381; 18403594 10.2353/ajpath.2008.070988 PMC2329846

[9] Q. Lin , Z. Chen , Z. L. Shen , F. Xue , J. J. Qin , X. P. Kang , Z. R. Chen , Z. Y. Xia , L. Gao , X. Z. Chen , Front. Oncol. 2022, 12, 776834. 36185204 10.3389/fonc.2022.776834 PMC9523251

[10] a) X. Chen , J. R. Cubillos‐Ruiz , Nature Reviews. Cancer 2020, 21, 71. 33214692 10.1038/s41568-020-00312-2 PMC7927882

[11] A. Walczak , K. Gradzik , J. Kabzinski , K. Przybylowska‐Sygut , I. Majsterek , Oxid. Med. Cell. Longev. 2019, 2019, 10.1155/2019/5729710. PMC6378054 30863482

[12] P. Walter , D. Ron , Science 2011, 334, 1081. 22116877 10.1126/science.1209038

[13] Y. Lin , M. Jiang , W. Chen , T. Zhao , Y. Wei , Biomedicine & pharmacotherapy = Biomedecine & pharmacotherapie 2019, 118, 109249. 31351428 10.1016/j.biopha.2019.109249

[14] a) C. Hetz , Nat. Rev. Mol. Cell Biol. 2012, 13, 2, 89; 10.1038/nrm3270 22251901

[15] C. A. L. L. T. M. H. A. s.‐K. , Mol. Cell 2018, 71, 629.30118681

[16] J. R., Cubillos‐Ruiz , S. E., Bettigole , L. H., Glimcher , Cell 2017, 168, 692. 28187289 10.1016/j.cell.2016.12.004 PMC5333759

[17] a) F. Reggiori , M. Molinari , Physiol. Rev. 2022, 102, 1393; 35188422 10.1152/physrev.00038.2021 PMC9126229

[18] a) J. F. Ehrmann , D. B. Grabarczyk , M. Heinke , L. Deszcz , R. Kurzbauer , O. Hudecz , A. Shulkina , R. Gogova , A. Meinhart , G. A. Versteeg , T. Clausen , Science 2023, 379, 1117; 36758105 10.1126/science.ade8873

[19] a) R. C. Russell , K. L. Guan , EMBO J. 2022, 41, 110031;

[20] J. Yong , J. D. Johnson , P. Arvan , J. Han , R. J. Kaufman , Nat. Rev. Endocrinol. 2021, 17, 455. 34163039 10.1038/s41574-021-00510-4 PMC8765009

[21] C. D. Zhu , Y. F. Xie , Q. Li , Z. W. Zhang , J. Chen , K. Zhang , X. F. Xia , D. L. Yu , D. Q. Chen , Z. Y. Yu , J. Chen , Drug Resist. Updates 2023, 68, 100933. 10.1016/j.drup.2023.100933 36821972

[22] S. J. Marciniak , J. E. Chambers , D. Ron , Nat. Rev. Drug Discovery 2022, 21, 115. 34702991 10.1038/s41573-021-00320-3

[23] a) S. Samanta , S. Yang , B. Debnath , D. Xue , Y. Kuang , K. Ramkumar , A. S. Lee , M. Ljungman , N. Neamati , Cancer Res. 2021, 81, 1883; 33531374 10.1158/0008-5472.CAN-20-1540 PMC8137563

[24] a) P. Zhang , X. Yan , X. Zhang , Y. Liu , X. Feng , Z. Yang , J. Zhang , X. Xu , Q. Zheng , L. Liang , H. Han , Circ. Res. 2023, 133, 739; 37750320 10.1161/CIRCRESAHA.123.322686

[25] S. Oyadomari , M. Mori , Cell Death Differ. 2004, 11, 381. 14685163 10.1038/sj.cdd.4401373

[26] a) S. A. Oakes , Am. J. Pathol. 2020, 190, 934, 32112719 10.1016/j.ajpath.2020.01.010 PMC7237829

[27] P. Jiao , W. Fan , X. Ma , R. Lin , Y. Zhao , Y. Li , H. Zhang , X. Jia , Y. Bi , X. Feng , M. Li , W. Liu , K. Zhang , L. Sun , Autophagy 2023, 19, 3113. 37482689 10.1080/15548627.2023.2238579 PMC10621274

[28] B. P. Jain , S. Pandey , N. Saleem , G. K. Tanti , S. Mishra , S. K. Goswami , Cell Stress Chaperones 2017, 22, 853. 28634818 10.1007/s12192-017-0816-7 PMC5655373

[29] F. Bray , M. Laversanne , H. Sung , J. Ferlay , R. L. Siegel , I. Soerjomataram , A. Jemal , Ca‐a Cancer J. Clinicians 2024, 74, 229. 10.3322/caac.21834 38572751

[30] W. Zhu , J. Li , R. Zhang , Y. Cai , C. Wang , S. Qi , S. Chen , X. Liang , N. Qi , F. Hou , EMBO J. 2019, 38, 102075. 10.15252/embj.2019102075 PMC6745499 31390091

[31] P. Nasarre , I. V. Bonilla , J. S. Metcalf , E. G. Hilliard , N. Klauber‐DeMore , Melanoma Res. 2018, 28, 185. 29553967 10.1097/CMR.0000000000000440

[32] a) D. J. Anderson , R. L. Moigne , S. Djakovic , B. Kumar , M. Rolfe , Cancer Cell 2015, 28, 653; 26555175 10.1016/j.ccell.2015.10.002 PMC4941640

[33] X. Yu , X.‐L. Teng , F. Wang , Y. Zheng , G. Qu , Y. Zhou , Z. Hu , Z. Wu , Y. Chang , L. Chen , H.‐B. Li , B. Su , L. Lu , Z. Liu , S.‐C. Sun , Q. Zou , J. Exp. Med. 2018, 215, 2463. 30115741 10.1084/jem.20180397 PMC6122976

[34] E. Suárez‐Martínez , S. R. Piersma , T. V. Pham , I. V. Bijnsdorp , C. R. Jimenez , A. Carnero , J. Exp. Clin. Cancer Res. 2024, 43, 150. 38807192 10.1186/s13046-024-03071-2 PMC11134651

[35] a) M. M. Yan , J. D. Ni , D. Song , M. Ding , J. Huang , Oncol. Lett. 2015, 10, 1959; 26622781 10.3892/ol.2015.3508 PMC4579870

[36] P. Y. Huang , S. Y. Liang , Y. Xiang , M. R. Li , M. R. Wang , L. H. Liu , Small 2024, 20, e231105. 10.1002/smll.202311056 38377262

[37] Y. Tang , M. Chen , L. Zhou , J. Ma , Y. Li , H. Zhang , Z. Shi , Q. Xu , X. Zhang , Z. Gao , Cell Discov. 2019, 5, 3. 30622739 10.1038/s41421-018-0077-3 PMC6323126

[38] a) U. Kück , D. Radchenko , I. Teichert , Biol. Chem. 2019, 400, 1005. 31042639 10.1515/hsz-2019-0173

[39] Y. Tang , G. Fang , F. Guo , H. Zhang , Z. Zhou , Cancer Cell 2020, 38, 115. 32589942 10.1016/j.ccell.2020.05.019

[40] a) G. K. Tanti , S. Pandey , S. K. Goswami , Biochem. Biophys. Res. Commun. 2015, 463, 524; 26022125 10.1016/j.bbrc.2015.05.069

[41] M. Deng , J. W. Tam , L. Wang , K. Liang , S. Li , L. Zhang , H. Guo , X. Luo , Y. Zhang , A. Petrucelli , B. K. Davis , B. J. Conti , W. J. Brickey , C.‐C. Ko , Y. L. Lei , S. Sun , J. P. Y. Ting , Nat. Commun. 2020, 11, 2193. 32366851 10.1038/s41467-020-16014-0 PMC7198545

[42] A. D. Garg , H. Maes , A. R. Van Vliet , P. Agostinis , Molecular Cellular Oncol. 2015, 2, e975089. 10.4161/23723556.2014.975089 PMC4905250 27308392

[43] J. S. Reis‐Filho , K. Savage , M. B. K. Lambros , M. James , D. Steele , R. L. Jones , M. Dowsett , Mod. Pathol. 2006, 19, 999. 16648863 10.1038/modpathol.3800621

