# Correction to “TRAF3IP3 Induces ER Stress‐Mediated Apoptosis with Protective Autophagy to Inhibit Lung Adenocarcinoma Proliferation”

**DOI:** 10.1002/advs.202513687

**Published:** 2025-09-06

Zhao G, Qi J, Li F, Ma H, Wang R, Yu X, Wang Y, Qin S, Wu J, Huang C, Ren H, Zhang B. Adv Sci (Weinh). 2025 May;12(17):e2411020.

In paragraph 1 of the “Results” section, the text “we detected the expression of TRAF3IP3 in tumor tissues versus matched normal tissues by conducting immunohistochemistry (IHC) analysis on 126 LUAD patients at our single center.” was incorrect. This should have read: we detected the expression of TRAF3IP3 in tumor tissues versus matched normal tissues by conducting immunohistochemistry (IHC) analysis on 63 LUAD patients at our single center.

The same typographical error appears in “Experimental Section,” Paragraph 1, the text “The clinicopathological characteristics of these 126 patients are provided in Table S1 (Supporting Information)” should be “The clinicopathological characteristics of these 63 patients are provided in Table S1 (Supporting Information)”

During the validation of the statistical tables, slight discrepancies were found between the submitted **Table** **1** and the recalculated dataset. Specifically: Formula implementation variances were made in 3 subcategories (smoking history, location, and Distant metastasis) and a typographical error in the subcategory of Tumor stage III.

**Table 1:** Upregulation of TRAF3IP3 correlates with Lung adenocarcinoma progression.

	TRAF3IP3 mRNA				*p* value (Fisher's Test)^a)^
Characteristic	Low expression	High expression	n	Increased [%]
**Age**, years					0.106
<60	13	21	34	61.8	
≥60	17	12	29	41.4	
**Sex**					0.923
Female	14	15	29	51.7	
Male	16	18	34	52.9	
**Smoking history**					0.866
Yes	17	18	35	51.4	
No	13	15	28	53.6	
**Differentiation**					0.022
Low	11	4	15	26.7	
Moderate or high	19	29	48	60.4	
**Lymph node metastasis**					0.018
None	12	23	35	65.7	
N1–N3	18	10	28	35.7	
**Location**					0.682
Peripheral	17	17	34	50	
Central	13	16	29	55.2	
**Pulmonary lobe**					0.030
Left	10	20	30	66.7	
Right	20	13	33	39.4	
**Tumor stage**					0.028
I	10	22	32	68.8	
II	11	7	18	38.9	
III	9	4	13	30.8	
**T stage**					<0.001
T1	4	22	26	84.6	
T2	22	11	33	33.3	
T3	4	0	4	0	
**Distant metastasis**					‐
M0	30	33	63	52.4	
M1–M3	0	0	0		

Fisher's exact test was used to test the association between the categorical variables.

^a)^ Statistically significant, *p* < 0.05.

We apologize for this error. The original dataset (Supporting Information File) remains unchanged and fully valid. All revisions do not affect the statistical significance of clinical correlations, experimental findings, or the manuscript's conclusions.

## Supporting information



Supporting Information

