# Supplementary material for: TRAF3IP3 Induces ER Stress‐Mediated Apoptosis with Protective Autophagy to Inhibit Lung Adenocarcinoma Proliferation
Source: Adv Sci (Weinh). 2025 Mar 11;12(17):2411020. doi: 10.1002/advs.202411020 (PMC12061266; doi:10.1002/advs.202411020)
Supplement: Supplementary file 1 — Supporting Information [file ADVS-12-2411020-s001.pdf]

ADVANCED SCIENCE
Open Access

Supporting Information

for *Adv. Sci.*, DOI 10.1002/advs.202411020

TRAF3IP3 Induces ER Stress-Mediated Apoptosis with Protective Autophagy to Inhibit Lung Adenocarcinoma Proliferation

Guang Zhao, Jun Qi, Fang Li, Haotian Ma, Rui Wang, Xiuyi Yu, Yufei Wang, Sida Qin, Jie Wu, Chen Huang, Hong Ren* and Boxiang Zhang**

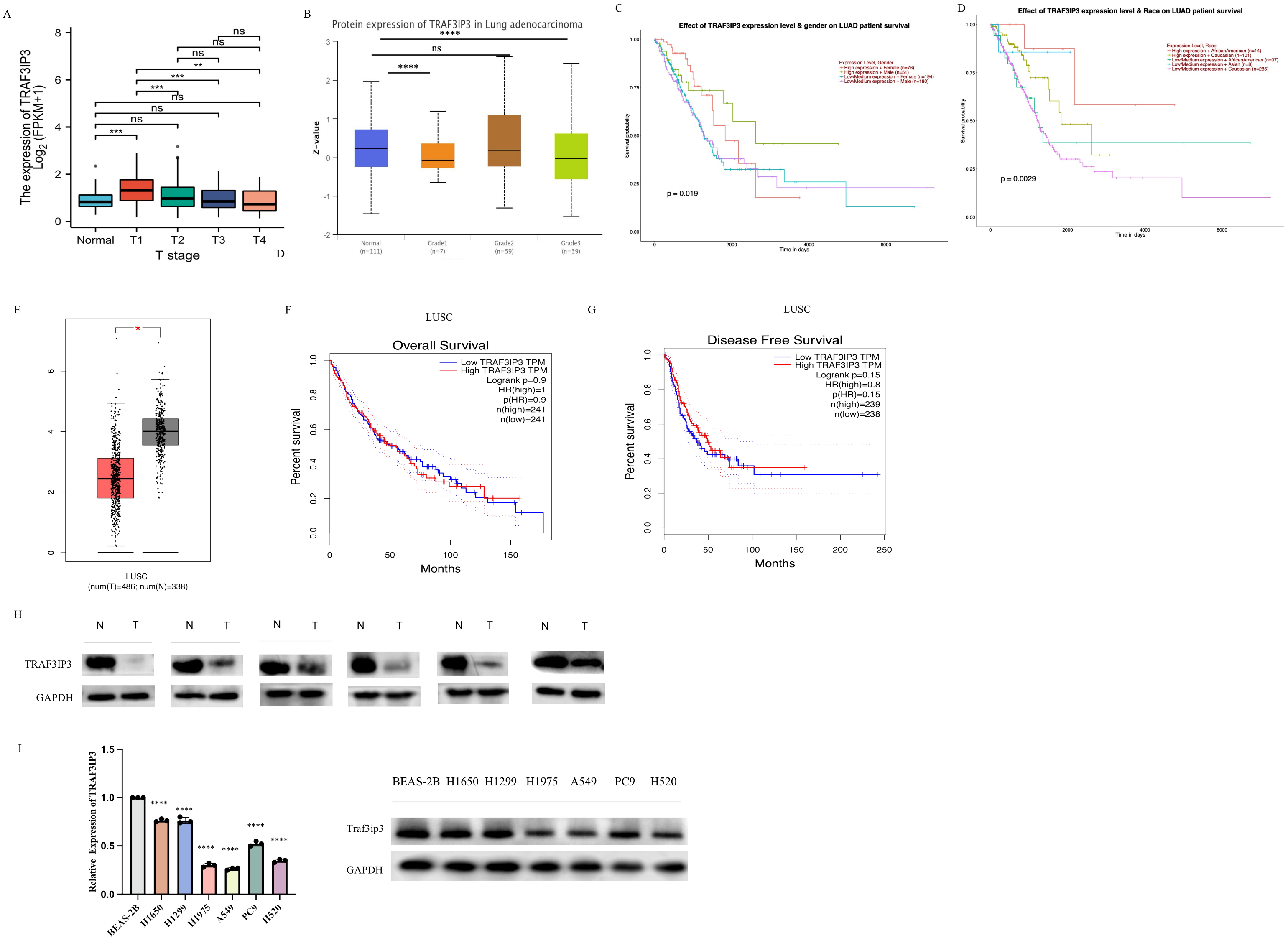

s-Figure 1 TRAF3IP3 expression level and its prognostic value in LUAD and LUSC

A The mRNA level of TRAF3IP3 in LUAD patients with normal, T1, T2, T3, and T4 tissues. **B** The protein level analysis of TRAF3IP3 in LUAD tumor differentiation grades. **C, D** The effect of TRAF3IP3 expression level and patients' gender and race on LUAD patient's survival by OS analysis. **D** Boxplots reveal that TRAF3IP3 mRNA levels are significantly downregulated in LUSC. **F, G** Kaplan-Meier survival analysis showed no significance in OS and DFS of LUSC patients with high and low expression of TRAF3IP3. **H** TRAF3IP3 expression in six LUAD tissues and matched adjacent non-tumor tissues was measured by western blotting analysis. **I** TRAF3IP3 mRNA and protein expression were analyzed by Western blotting in normal lung epithelial cells (BEAS-2B) and different lung cancer cell lines (A549, PC9, H1299, H1975, H1650, H520). ns: $P \geq 0.05$, $^{**}P < 0.01$, $^{***}P < 0.001$, $^{****}P < 0.0001$.

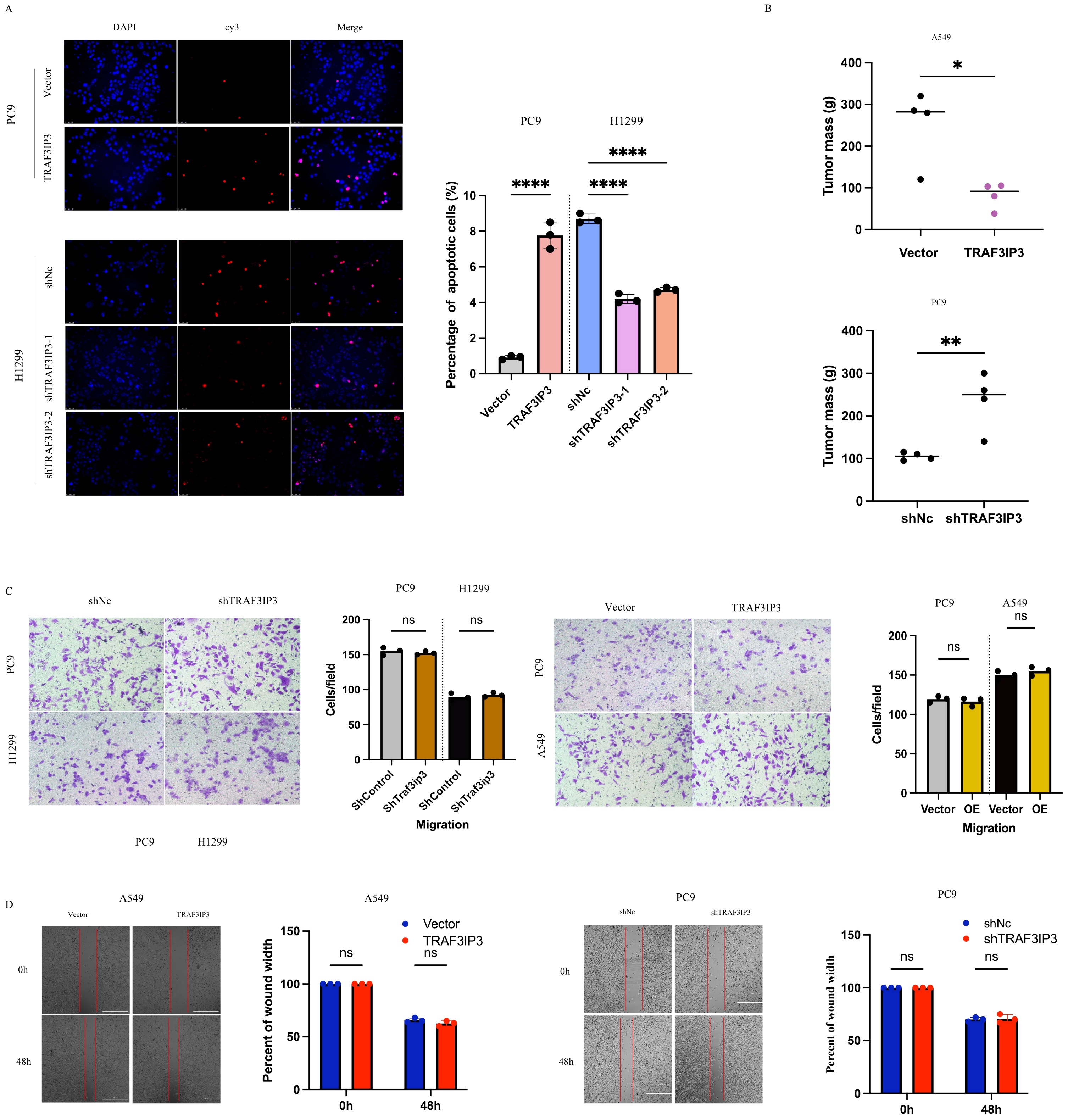

S-Figure 2 The effect of TRAF3IP3 on LUAD migration capability in vitro

A Western blotting was conducted to confirm the transfection efficiency of shtraf3ip3 in PC9 and H1299 cells. **B** Cell proliferation detection of PC9 and H1299 TRAF3IP3-knocking down cells was measured using TUNEL assay. Scale bar, 50μm. **C** The tumors of the xenografts were weighed and statistically analyzed. **D** The effect of TRAF3IP3 on the migration of LUAD cells was determined using a transwell assay. **E** The effect of TRAF3IP3 on LUAD cell migration was determined using a wound healing assay. At 0h and 48h after wound formation, the percentage of wound width was measured. B, C (n=4), D, E unpaired t-test and one-way ANOVA, n=3, median ± SD, ns: P ≥ 0.05, *P < 0.05, ****P < 0.0001.

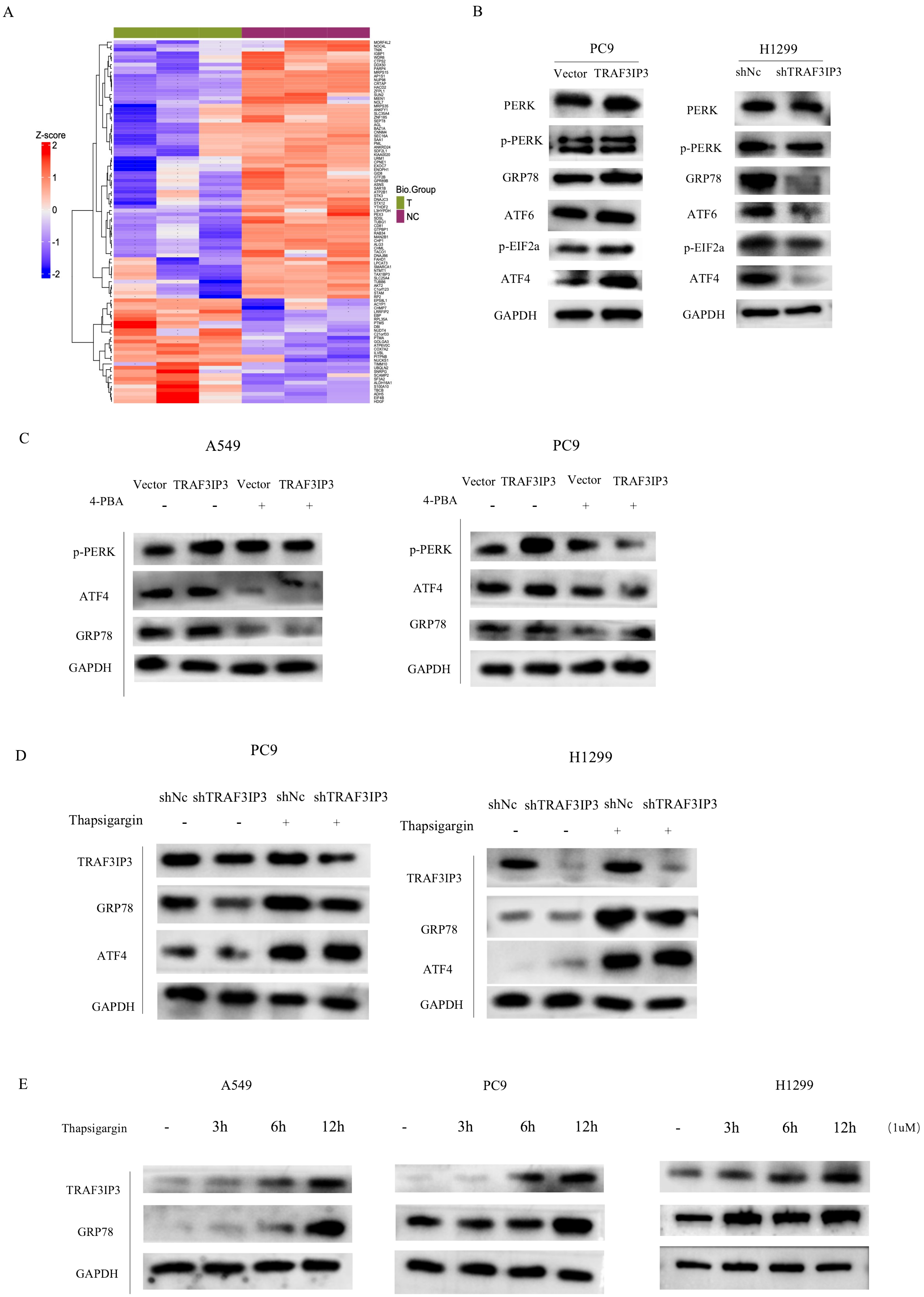
A
Z-score
2
1
0
-1
-2
Bio.Group
T
NC
B
PC9
Vector TRAF3IP3
H1299
shNc shTRAF3IP3
PERK
p-PERK
GRP78
ATF6
p-EIF2a
ATF4
GAPDH
C
A549
Vector TRAF3IP3 Vector TRAF3IP3
4-PBA - - + +
p-PERK
ATF4
GRP78
GAPDH
PC9
Vector TRAF3IP3 Vector TRAF3IP3
4-PBA - - + +
p-PERK
ATF4
GRP78
GAPDH
D
PC9
shNc shTRAF3IP3 shNc shTRAF3IP3
Thapsigargin - - + +
TRAF3IP3
GRP78
ATF4
GAPDH
H1299
shNc shTRAF3IP3 shNc shTRAF3IP3
Thapsigargin - - + +
TRAF3IP3
GRP78
ATF4
GAPDH
E
A549
PC9
H1299
Thapsigargin - 3h 6h 12h - 3h 6h 12h - 3h 6h 12h (1uM)
TRAF3IP3
GRP78
GAPDH

S-Figure 3 TRAF3IP3 activates ER stress and induces PERK and ATF4 in LUAD cells

A Heat map displaying the significantly differentially expressed proteins between control and TRAF3IP3-overexpression cells. **B** Western blots showed ER stress-related proteins, including p-PERK, PERK, ATF6, GRP78, p-EIF2a, ATF4, and GAPDH protein levels in PC9 TRAF3IP3-overexpression and H1299 TRAF3IP3-knocking down cell lines. **C** A549 and PC9 cells with TRAF3IP3 overexpression or with a control vector were treated with 1 mM 4-PBA (ER stress inhibitor) for 18 h. Immunoblot analysis was performed with the indicated antibodies. **D** PC9 and H1299 cells with ShNC and ShTRAF3IP3 were treated with 1 uM thapsigargin for 18 h. Immunoblot analysis was performed with the indicated antibodies. **E** Western blotting detection of TRAF3IP3 and GRP78 expression in A549, PC9, and H1299 cells treated with 1um thapsigargin for time course analysis using GAPDH as an internal reference.

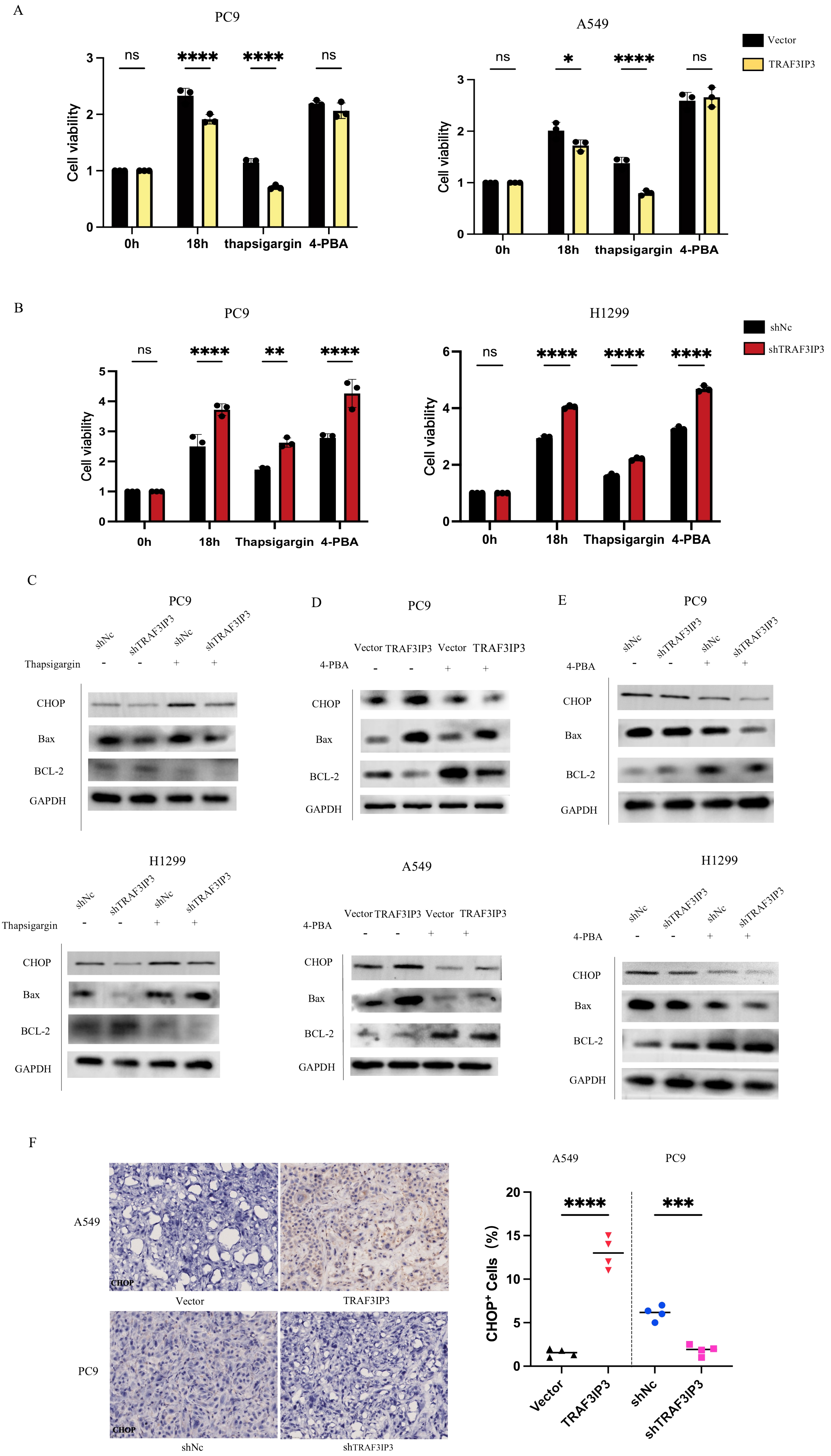
A
PC9
A549
Vector
TRAF3IP3
ns
****
*
Cell viability
0h
18h
thapsigargin
4-PBA
B
PC9
H1299
shNc
shTRAF3IP3
**
Thapsigargin
C
D
E
Thapsigargin
4-PBA
CHOP
Bax
BCL-2
GAPDH
A549
F
CHOP
Vector
TRAF3IP3
shNc
shTRAF3IP3
***
CHOP⁺ Cells (%)

S-Figure 4 TRAF3IP3 depletion inhibits ER stress-related apoptosis

A A549 and PC9 cells with TRAF3IP3 overexpression or with a control vector were cultured for 18 h and then treated with 1 uM thapsigargin for another 18 h. CCK8 assay was performed to assess the cell viability. **B** PC9 and H1299 cells with ShNc or ShTRAF3IP3 were cultured for 18 h and then treated with 1 mM 4-PBA for another 18 h. CCK8 assay was performed to assess the cell viability. **C** PC9 and H1299 cells with ShNc or Sh TRAF3IP3 were treated with 1 uM thapsigargin for 18 h. Immunoblot analysis was performed to detect apoptosis-related proteins including Bax, Bcl-2 and CHOP levels. **D** A549 and PC9 cells with TRAF3IP3 overexpression or with a control vector were treated with 1 mM 4-PBA for 18 h. Immunoblot analysis was performed to detect apoptosis-related proteins, as mentioned above. **E** PC9 and H1299 cells with ShNc or Sh TRAF3IP3 were treated with 1 mM 4-PBA for 18 h. Apoptosis-related proteins were analyzed by Immunoblot analysis. A, B, F unpaired t-test and one-way ANOVA, n=4, median ±SD, ns, $P \geq 0.05$, $^{*}P < 0.05$, $^{**}P < 0.005$, $^{****}P < 0.0001$.

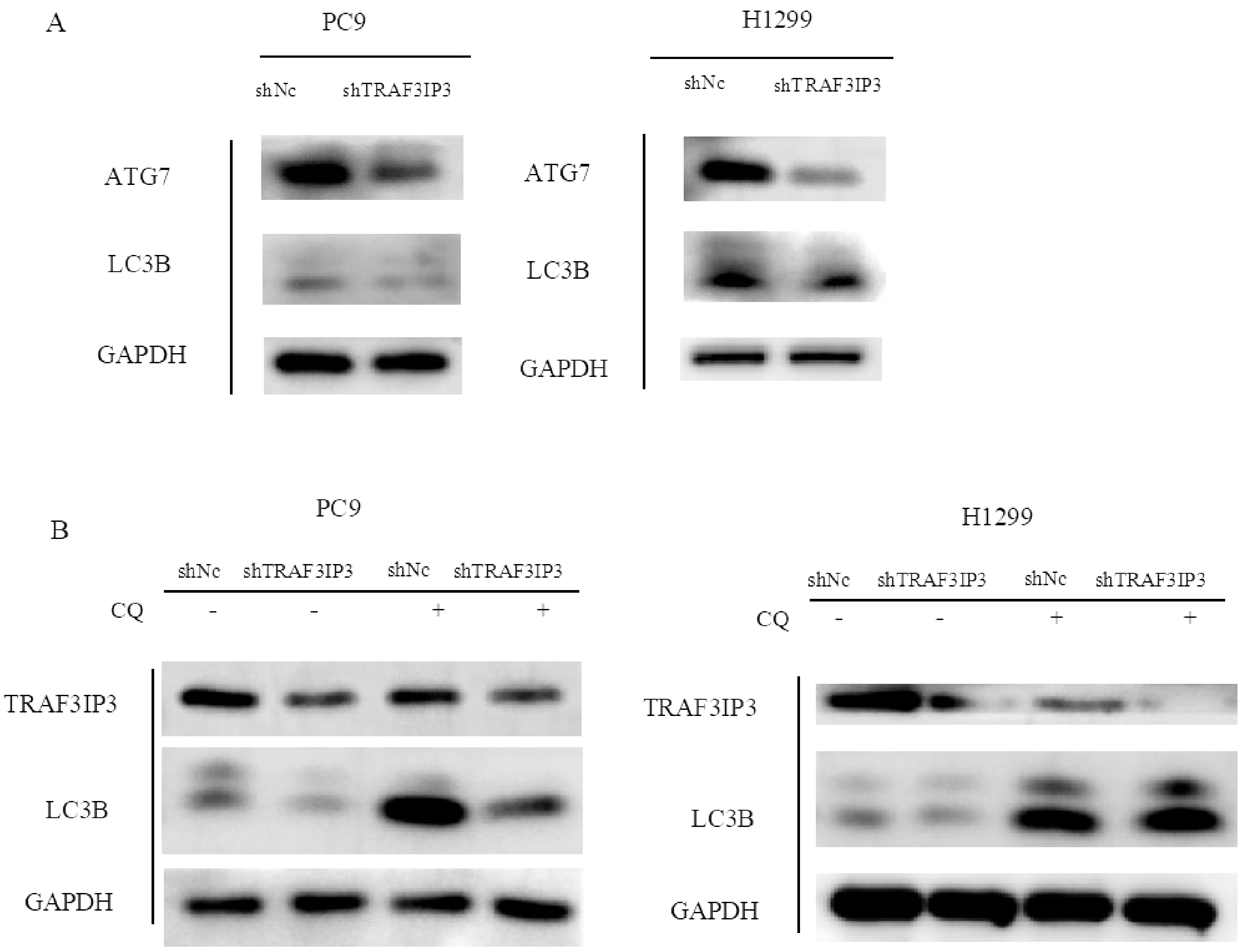

S-Figure 5

In PC9 and H1299 cells with stable TRAF3IP3 knockdown or control, (**A**) western blotting was performed to detect autophagy-related proteins (LC3B, ATG7); and (**B**) the cells were subjected to the treatment with CQ (20 μM for 20 h) or not, the protein levels of LC3 were examined.

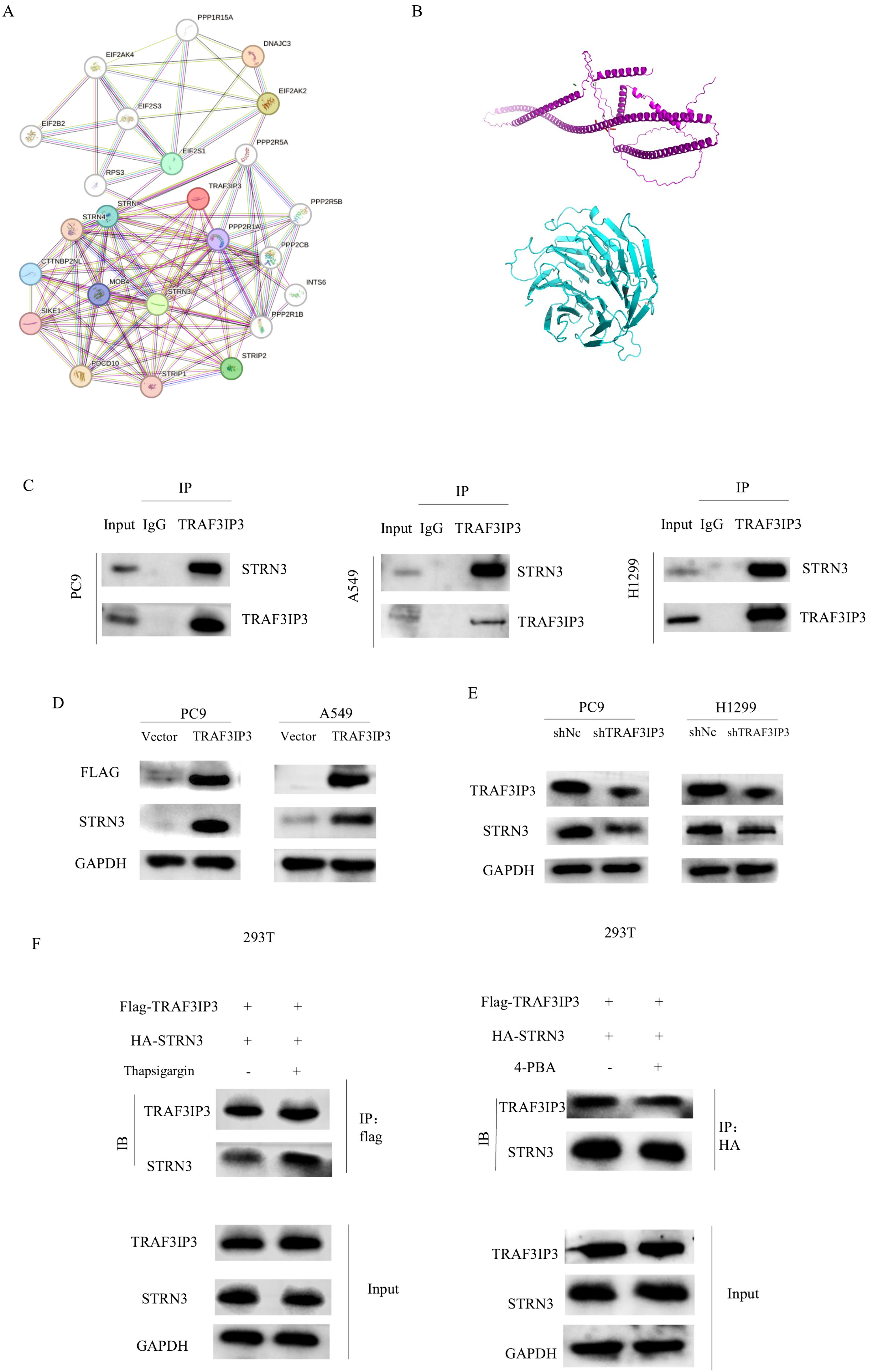
A
PPP1R15A
DNAJC3
EIF2AK4
EIF2AK2
EIF2S3
EIF2B2
EIF2S1
PPP2R5A
RPS3
TRAF3IP3
STRN
PPP2R5B
STRN4
PPP2R1A
PPP2CB
CTTNBP2NL
MOB4
INTS6
STRN3
SIKE1
PPP2R1B
STRIP2
PDCD10
STRIP1
B
C
IP
Input IgG TRAF3IP3
PC9
STRN3
TRAF3IP3
A549
H1299
D
PC9
A549
Vector TRAF3IP3
FLAG
STRN3
GAPDH
E
PC9
H1299
shNc shTRAF3IP3
TRAF3IP3
STRN3
GAPDH
F
293T
Flag-TRAF3IP3 + +
HA-STRN3 + +
Thapsigargin - +
IB
TRAF3IP3
STRN3
IP：flag
Input
GAPDH
4-PBA - +
IP：HA

S-Figure 6 TRAF3IP3 interacts with STRN3

A The protein-protein interaction network of TRAF3IP3 was analyzed using the STRING database. **B** Tertiary structure of TRAF3IP3 (purple chain) and STRN3 (sky-blue chain). **C, D** STRN3 expression changes after TRAF3IP3 overexpression/ knockdown in A549, PC9 and H1299 cells were detected using western blotting, with GAPDH as control. **E** endogenous reciprocal co-IP of TRAF3IP3 and STRN3 in A549, PC9 and H1299 cell lines. IgG was used as a negative control. **F** Co-IP was performed on 293T cell lysates overexpressing Flag- TRAF3IP3 and HA-STRN3 to observe protein interaction affinity in the presence of thapsigargin or 4-PBA.

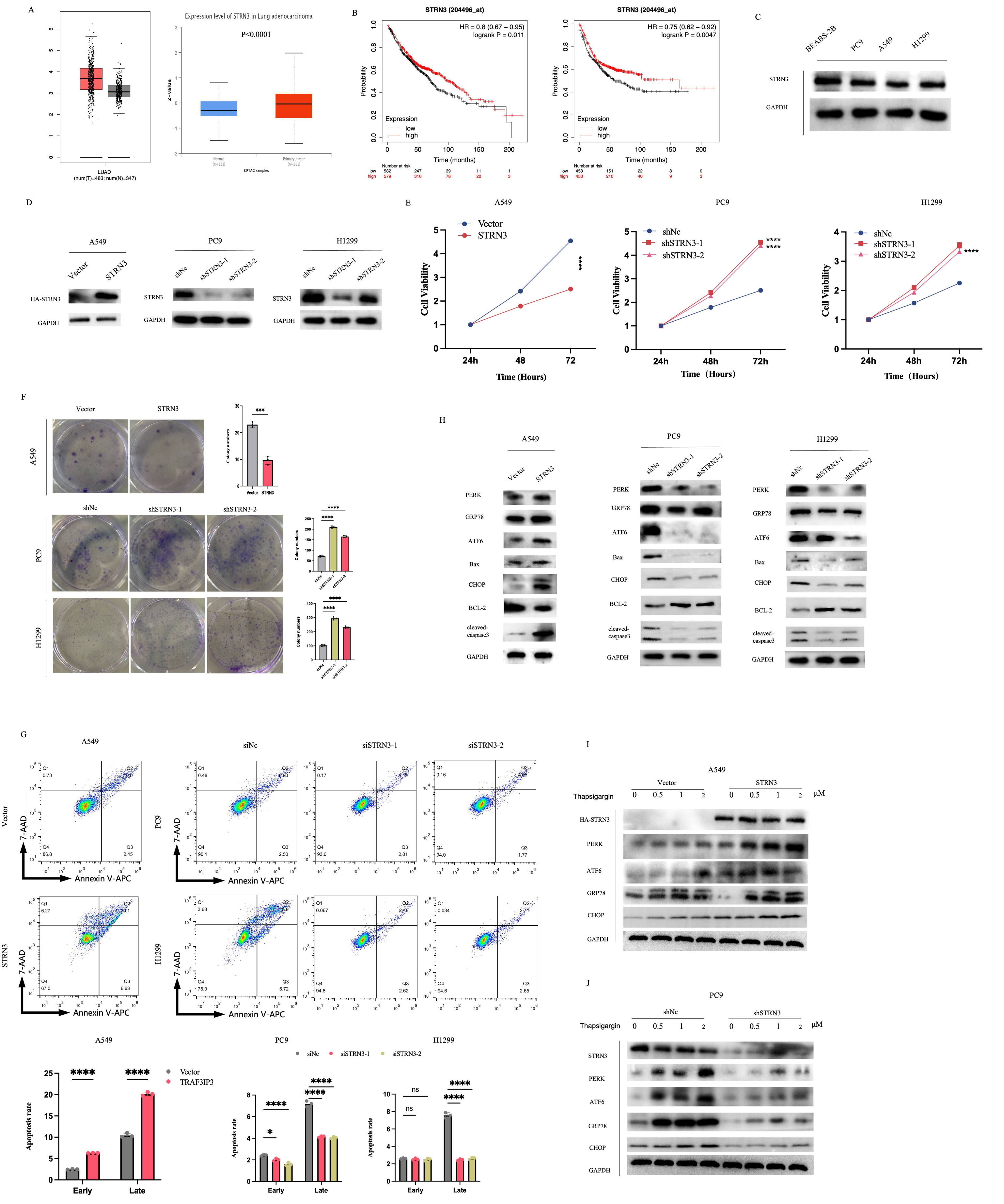

A
LUAD
(num(T)=483; num(N)=347)
Expression level of STRN3 in Lung adenocarcinoma
P<0.0001
Z-value
Normal (n=111)
Primary tumor (n=111)
CPTAC samples
B
STRN3 (204496_at)
HR = 0.8 (0.67 − 0.95)
logrank P = 0.011
HR = 0.75 (0.62 − 0.92)
logrank P = 0.0047
Probability
Expression
low
high
Time (months)
Number at risk
low 582 247 39 11 1
high 579 316 78 20 3
low 453 151 22 8 0
high 453 210 40 9 3
C
BEAS-2B
PC9
A549
H1299
STRN3
GAPDH
D
A549
Vector
STRN3
HA-STRN3
GAPDH
PC9
shNc
shSTRN3-1
shSTRN3-2
STRN3
GAPDH
H1299
E
A549
Vector
STRN3
Cell Viability
Time (Hours)
24h
48
72
PC9
shNc
shSTRN3-1
shSTRN3-2
48h
72h
Time（Hours)
H1299
F
Vector
STRN3
A549
Colony numbers
shNc
shSTRN3-1
shSTRN3-2
PC9
H1299
H
A549
PERK
GRP78
ATF6
Bax
CHOP
BCL-2
cleaved-caspase3
GAPDH
PC9
H1299
G
A549
siNc
siSTRN3-1
siSTRN3-2
Vector
STRN3
PC9
H1299
7-AAD
Annexin V-APC
Apoptosis rate
Early
Late
Vector
TRAF3IP3
siNc
siSTRN3-1
siSTRN3-2
ns
I
A549
Thapsigargin
Vector
STRN3
0 0.5 1 2 0 0.5 1 2 μM
HA-STRN3
PERK
ATF6
GRP78
CHOP
GAPDH
J
PC9
shNc
shSTRN3
STRN3

S-Figure 7 STRN3 regulates LUAD cell apoptosis and ER stress

A mRNA and protein expression level of STRN3 in normal tissues and LUAD tissues. **B** OS and DFS in LUAD patients with high and low STRN3 expression levels were obtained from the K-M analysis. **C** Western blotting was used to examine STRN3 expression in normal lung epithelial cells (BEAS-2B) and lung cancer cells (A549/PC9/H1299). **D** Validation of STRN3 overexpression or knockdown transfection efficiency in indicated cell lines. **E, F** CCK8 assay and colony formation assay was used to determine the effect of overexpressing or knocking down STRN3 on LUAD cell proliferation. **G** The effect of STRN3 overexpression or knockdown on cell apoptosis in A549, PC9 and H1299 cells was detected by flow cytometry. **H** The effect of altering TRAF3IP3 expression on the protein levels of molecules related to cell apoptosis and ER stress was assessed using western blotting in A549, PC9, and H1299 cell lines. **I** Western blots of ER stress-related protein levels in control and STRN3 overexpressing A549 cells treated with thapsigargin. **J** Western blots of ER stress-related protein levels in control and STRN3 knockdown A549 cells treated with thapsigargin. E (n=4), F, G (n=3) unpaired t-test and one-way ANOVA, median ± SD, ns: $P \geq 0.05$, ***$P < 0.001$, ****$P < 0.0001$.

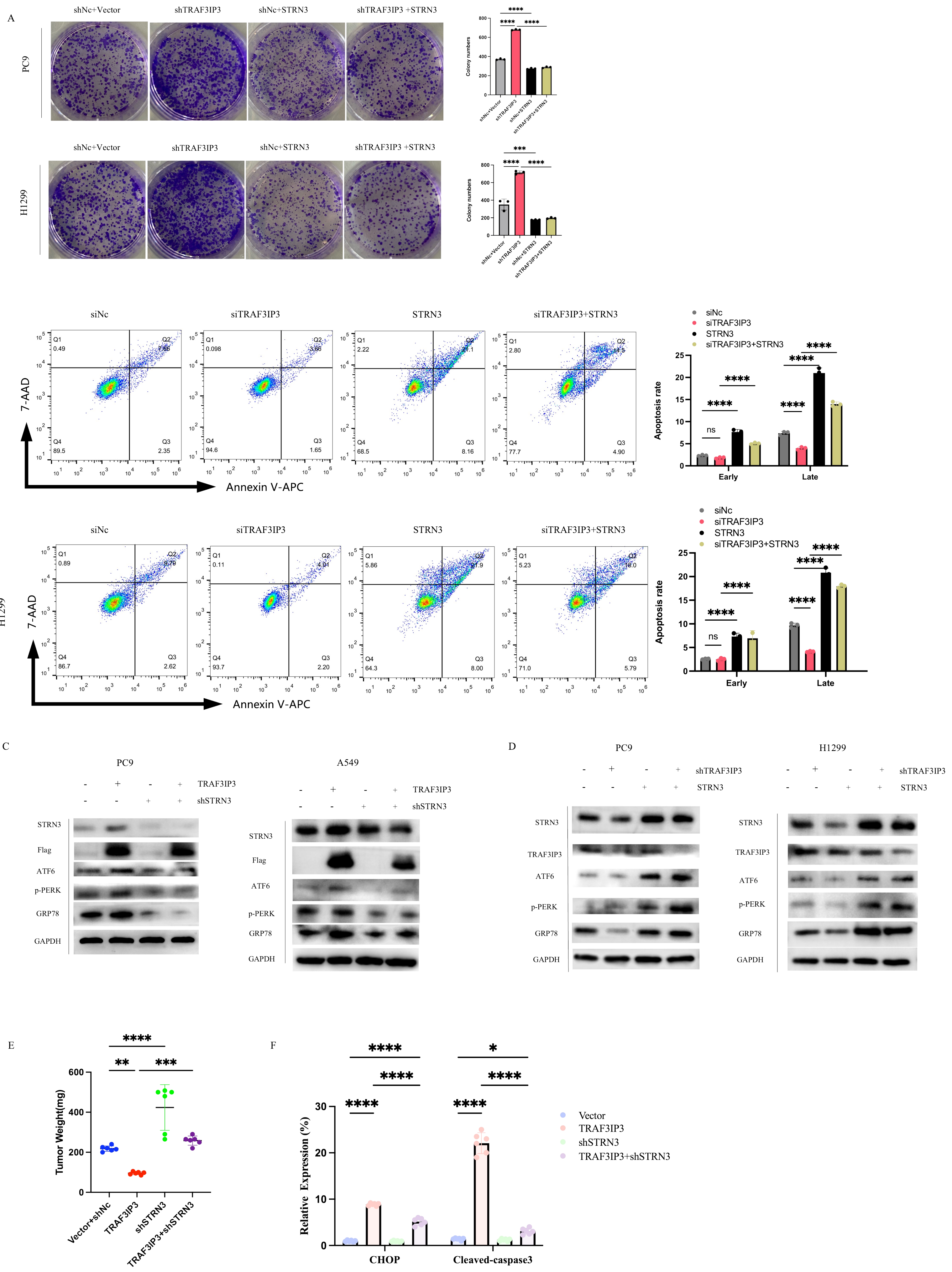
A
shNc+Vector
shTRAF3IP3
shNc+STRN3
shTRAF3IP3 +STRN3
PC9
H1299
Colony numbers
shNc+Vector
shTRAF3IP3
shNc+STRN3
shTRAF3IP3+STRN3
B
siNc
siTRAF3IP3
STRN3
siTRAF3IP3+STRN3
7-AAD
Annexin V-APC
Apoptosis rate
Early
Late
ns
C
PC9
A549
TRAF3IP3
shSTRN3
STRN3
Flag
ATF6
p-PERK
GRP78
GAPDH
D
PC9
H1299
shTRAF3IP3
STRN3
STRN3
TRAF3IP3
ATF6
p-PERK
GRP78
GAPDH
E
Tumor Weight(mg)
Vector+shNc
TRAF3IP3
shSTRN3
TRAF3IP3+shSTRN3
F
Relative Expression (%)
CHOP
Cleaved-caspase3
Vector
TRAF3IP3
shSTRN3
TRAF3IP3+shSTRN3

S-Figure 8 TRAF3IP3 regulates ER stress-related apoptosis through STRN3

A The effect of vector+shnc, vector+shTRAF3IP3, over-STRN3+shnc, and over-STRN3+ shTRAF3IP3 on cell colony formation capability in PC9 and H1299 cells was assessed using colony formation assay. **B** Flow cytometry was performed to determine the effect of vector+control, vector+siTRAF3IP3, over-STRN3+control, and over-STRN3+siTRAF3IP3 treatment on cell apoptosis of PC9 and H1299 cells. **C, D** Immunoblot analysis of cell lysates in each group were performed to detect ER stress-related molecular levels with indicated antibodies. **E** The specified tumors of the xenografts were weighed and statistically analyzed. **F** Quantification of immunohistochemical staining in A549 positive xenograft tumor cells. A, B (n=3), E, F (n=6) unpaired t-test and two-way ANOVA, median ± SD, ns: $P \geq 0.05$, ***$P < 0.001$, ****$P < 0.0001$.

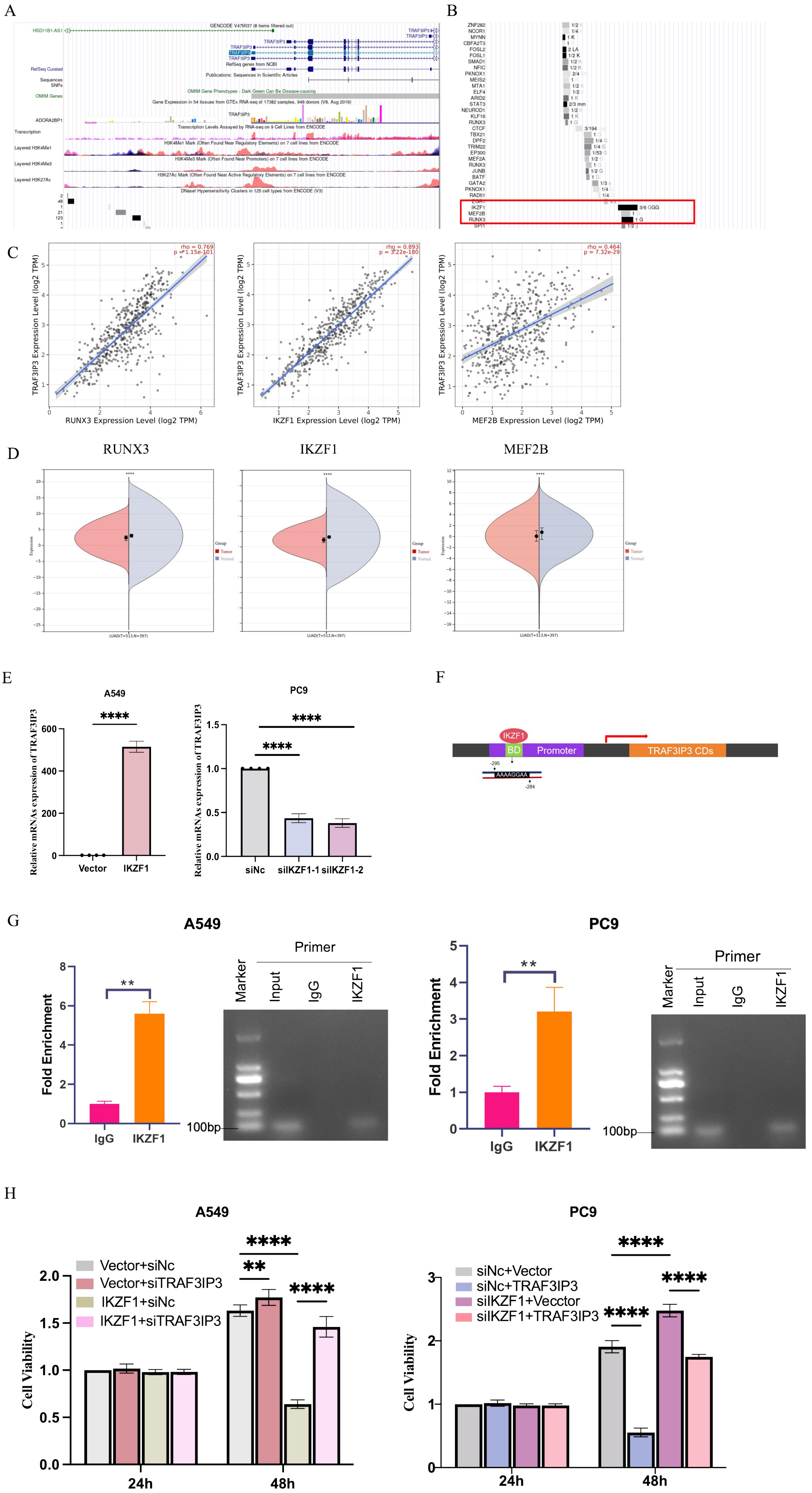
A
GENCODE V47lift37 (8 items filtered out)
HSD11B1-AS1
TRAF3IP3
RefSeq Curated
RefSeq genes from NCBI
Publications: Sequences in Scientific Articles
Sequences
SNPs
OMIM Genes
OMIM Gene Phenotypes - Dark Green Can Be Disease-causing
Gene Expression in 54 tissues from GTEx RNA-seq of 17382 samples, 948 donors (V8, Aug 2019)
ADORA2BP1
Transcription
Transcription Levels Assayed by RNA-seq on 9 Cell Lines from ENCODE
Layered H3K4Me1
H3K4Me1 Mark (Often Found Near Regulatory Elements) on 7 cell lines from ENCODE
Layered H3K4Me3
H3K4Me3 Mark (Often Found Near Promoters) on 7 cell lines from ENCODE
Layered H3K27Ac
H3K27Ac Mark (Often Found Near Active Regulatory Elements) on 7 cell lines from ENCODE
DNaseI Hypersensitivity Clusters in 125 cell types from ENCODE (V3)
B
ZNF282
NCOR1
MYNN
CBFA2T3
FOSL2
FOSL1
SMAD1
NFIC
PKNOX1
MEIS2
MTA1
ELF4
ARID2
STAT3
NEUROD1
KLF16
RUNX3
CTCF
TBX21
DPF2
TRIM22
EP300
MEF2A
RUNX3
JUNB
BATF
GATA2
PKNOX1
RAD51
IKZF1
MEF2B
RUNX3
SPI1
3/6 GGG
C
rho = 0.769
p = 1.15e-101
TRAF3IP3 Expression Level (log2 TPM)
RUNX3 Expression Level (log2 TPM)
rho = 0.893
p = 3.22e-180
IKZF1 Expression Level (log2 TPM)
rho = 0.464
p = 7.32e-29
MEF2B Expression Level (log2 TPM)
D
RUNX3
IKZF1
MEF2B
Expression
Group
Tumor
Normal
LUAD(T=513,N=397)
E
A549
PC9
Relative mRNAs expression of TRAF3IP3
Vector
IKZF1
siNc
siIKZF1-1
siIKZF1-2
F
IKZF1
BD
Promoter
TRAF3IP3 CDs
-295
AAAAGGAA
-284
G
A549
PC9
Fold Enrichment
IgG
IKZF1
Primer
Marker
Input
IgG
IKZF1
100bp
H
A549
PC9
Cell Viability
Vector+siNc
Vector+siTRAF3IP3
IKZF1+siNc
IKZF1+siTRAF3IP3
siNc+Vector
siNc+TRAF3IP3
siIKZF1+Vecctor
siIKZF1+TRAF3IP3
24h
48h

S-Figure 9 TRAF3IP3 transcription may be regualted by candidate transcription factors
A, B Possible upstream transcription factors through UCSC database (https://genome.ucsc.edu/). **C** **S**ignificant positive correlation between TRAF3IP3 and the expression of the three transcription factors were analyzed through TIMER online database (http://timer.cistrome.org/). **D** Boxplots reveal that RUXN3, IKZF1 and MEF2B mRNA levels are significantly downregulated in LUAD. **E** TRAF3IP3 mRNA levels in IKZF1-knockdown A549 and PC9 cells were measured by qRT-PCR. **F** Schematic of primer design for ChIP-qRT-PCR in the binding site between IKZF1 and TRAF3IP3 promoter region. **G** Using ChIP-qRT-PCR and agarose gel electrophoresis, the binding relationship between IKZF1 and the promoter region of TRAF3IP3 was confirmed. **H** The CCK-8 assay was performed to determine the effect of the indicated groups on the cell viability of the LUAD cells. E (n=5), G (n=3) one-way ANOVA; H(n=5) two-way ANOVA, $^{**}P < 0.01$, $^{****}P < 0.0001$.

Table S1 The TRAF3IP3 expression in LUAD and adjacent normal lung tissues.

	TRAF3IP3				
	Low	High	Total	χ^2	*P* value
Normal	15	48	63	25.047	<0.001
Tumor	43	20	63		

Table S2 Antibodies, inhibitors, RT-PCR primers, shRNAs and siRNAs used in this study

Antibodies and inhibitors information in this study

Antibodies	Company	Catalog No
GAPDH	Abclonal	A19056
TRAF3IP3	Santa Cruz Biotechnology	sc-398781
E-Cadherin	Cell Signaling Technology	14472
N-Cadherin	Cell Signaling Technology	5741
Lamin-B1	Proteintech	12987-1-AP
STRN3	Abclonal	A6756
STRN3	Proteintech	23966-1-AP
Flag	Proteintech	66008-4-Ig
HA	Proteintech	51064-2-AP
HSP90	Proteintech	4877
PERK	Proteintech	20582-1-AP
p-PERK	Cell Signaling Technology	3179
ATF4	Cell Signaling Technology	11815
GRP78	Cell Signaling Technology	3177
CHOP	Cell Signaling Technology	2895T
ATF6	Proteintech	66563-1-Ig
p-EIF2α	Cell Signaling Technology	3398
IRE1	Cell Signaling Technology	3294
LC3B	Cell Signaling Technology	2775
ATG7	Abclonal	A19604
Cleaved Caspase 3	Proteintech	25128-1-AP
Bax	Cell Signaling Technology	2772
Bcl-2	Cell Signaling Technology	15071
Goat Anti-Rabbit IgG(H+L)	Proteintech	SA00001-2
Goat Anti-mouse IgG(H+L)	Proteintech	SA00001-1
Mouse Anti-Rabbit IgG, Light Chain	Proteintech	SA00001-7L
Rabbit Anti-Mouse IgG Kappa Light Chain	Proteintech	SA00001-19
Goat Anti-Rabbit IgG (H+L) Alexa Fluor 594	Abways	AB0518
Goat Anti-Mouse IgG (H+L) Alexa Fluor 488	Abways	AB0142
Thapsigargin	MCE	HY-13433

4-PBA (4-Phenylbutyric acid)	MCE	HY-A0281
Chloroquine (CQ)	Sigma-Aldrich	C6628
Nec-1 (Necrostatin-1)	Selleck	S8037
Z-VAD-FMK	Selleck	S7023
Fer-1 (Ferrostatin-1)	Selleck	S-7243

Primer information in this study

Primer names	Sequence (5′-3′)
GAPDH F	GGAGCGAGATCCCTCCAAAAT
GAPDH R	GGCTGTTGTCATACTTCTCATGG
TRAF3IP3 F	ACCCAGGACCTACAAGATCAAC
TRAF3IP3 R	GTTAGGGAAGGACTTTGGACTC
STRN3 F	TCTGGAGGAGGCAAGTCATTTAT
STRN3 R	GGTATGGTCCTCAGAAGCAGTAA

Sequences of lentiviral shRNAs and interference oligonucleotides

Target gene	Target sequence (5′-3′)
shNc	TTCTCCGAACGTGTCACGT
shTRAF3IP3-1	GAACCCTTGTCTTGTGCCAGAA
shTRAF3IP3-2	GGGAGCTGGTCCTAAAACA
shSTRN3	CCTATCTACACATTTAGGGTTCAAG
siNc	UUCUCCGAACGUGUCACGU TT
siTRAF3IP3-1	GGCAGAAGGACCAACAAUU
siTRAF3IP3-2	GGGAGCUGGUCCUAAAACA
siSTRN3-1	CCUAUCUACACAUUUAGGG
siSTRN3-2	AGAUCCUAAUGGAAUCUAUUU
siPERK-1	GAUAGUGACGAA
siPERK-2	CCUCAAGCCAUCCAACAUAUU
siATF4-1	GCCUAGGUCUCUUAGAUGA
siATF4-2	CCAGAUCAUUCCUUUAGUUUA
siATG7-1	GAGAUAUGGGAAUCCAUAATT
siATG7-2	GCCUGCUGAGGAGCUCUCCA

Table S3 online databases used in the work.

Database	Online link
Kaplan-Meier Plotter	https://kmplot.com/analysis/
GEPIA	http://gepia.cancer-pku.cn/index.html
UALCAN	https://ualcan.path.uab.edu/

Genomic Data Commons	https://gdc.cancer.gov/
STRING	https://string-db.org/

Table S4 Results of the H-dock analysis of TRAF3IP3 and STRN3

	model1	model2	model3	model4	model5	model6	model7	model8	model9	model10
Binding energy	-392.71	-370.24	-363.17	-358.53	-343.3	-342.4	-340.3	-339.99	-339.39	-337.85
RMSD	254.54	226.62	170.93	213.2	260.71	172.83	175.2	208.73	205.62	256.62

RMSD: root mean square deviation. Position differences of initial configurations.

Table S5 Details of the binding energy of interaction of model 1

name	distance	category	type	from	from chemistry	to	to chemistry
S:HIS562:ND1 - A:GLU114:OE2	3.09983	Electrostatic	Attractive Charge	S:HIS562:ND1	Positive	A:GLU114:OE2	Negative
S:LYS627:NZ - A:GLU114:OE2	5.52769	Electrostatic	Attractive Charge	S:LYS627:NZ	Positive	A:GLU114:OE2	Negative
A:GLY118:H - S:LEU583:O	3.04462	Hydrogen Bond	Conventional Hydrogen Bond	A:GLY118:H	H-Donor	S:LEU583:O	H-Acceptor
A:GLN313:HE22 - S:ASP503:OD1	1.74888	Hydrogen Bond	Conventional Hydrogen Bond	A:GLN313:HE22	H-Donor	S:ASP503:OD1	H-Acceptor
A:ARG522:HH22 - S:TYR443:OH	2.59019	Hydrogen Bond	Conventional Hydrogen Bond	A:ARG522:HH22	H-Donor	S:TYR443:OH	H-Acceptor
A:ARG522:HH22 - S:THR444:O	2.68975	Hydrogen Bond	Conventional Hydrogen Bond	A:ARG522:HH22	H-Donor	S:THR444:O	H-Acceptor
S:THR497:HG1 - A:CYS524:SG	1.86549	Hydrogen Bond	Conventional Hydrogen Bond	S:THR497:HG1	H-Donor	A:CYS524:SG	H-Acceptor
A:SER111:HA - S:GLU578:OE1	2.35491	Hydrogen Bond	Carbon Hydrogen Bond	A:SER111:HA	H-Donor	S:GLU578:OE1	H-Acceptor
A:SER111:HB3 - S:GLU578:OE1	2.5768	Hydrogen Bond	Carbon Hydrogen Bond	A:SER111:HB3	H-Donor	S:GLU578:OE1	H-Acceptor
A:PRO112:HD2 - S:GLU578:OE1	2.88059	Hydrogen Bond	Carbon Hydrogen Bond	A:PRO112:HD2	H-Donor	S:GLU578:OE1	H-Acceptor
A:GLN302:HA - S:ASN542:OD1	3.08795	Hydrogen Bond	Carbon Hydrogen Bond	A:GLN302:HA	H-Donor	S:ASN542:OD1	H-Acceptor
A:GLU114:OE2 - S:HIS562	2.72376	Electrostatic	Pi-Anion	A:GLU114:OE2	Negative	S:HIS562	Pi-Orbitals
A:ARG107 - S:LYS534	4.92209	Hydrophobic	Alkyl	A:ARG107	Alkyl	S:LYS534	Alkyl
A:PRO112 - S:PRO560	4.84138	Hydrophobic	Alkyl	A:PRO112	Alkyl	S:PRO560	Alkyl

A:CYS524 - S:LEU494	4.67475	Hydrophobic	Alkyl	A:CYS524	Alkyl	S:LEU494	Alkyl
S:ARG446 - A:PRO519	4.97567	Hydrophobic	Alkyl	S:ARG446	Alkyl	A:PRO519	Alkyl
S:ALA561 - A:PRO112	3.93668	Hydrophobic	Alkyl	S:ALA561	Alkyl	A:PRO112	Alkyl
S:TYR489 - A:ARG522	4.98694	Hydrophobic	Pi-Alkyl	S:TYR489	Pi-Orbitals	A:ARG522	Alkyl
